# Tight Adherence (Tad) Pilus Genes Indicate Putative Niche Differentiation in Phytoplankton Bloom Associated *Rhodobacterales*

**DOI:** 10.3389/fmicb.2021.718297

**Published:** 2021-08-10

**Authors:** Ashley Isaac, Ben Francis, Rudolf I. Amann, Shady A. Amin

**Affiliations:** ^1^Marine Microbial Ecology Laboratory, Biology Program, New York University Abu Dhabi, Abu Dhabi, United Arab Emirates; ^2^Max Planck Institute for Marine Microbiology, Bremen, Germany

**Keywords:** Roseobacter, phytoplankton-bacteria interactions, phycosphere colonization, diatoms, microbiome

## Abstract

The multiple interactions of phytoplankton and bacterioplankton are central for our understanding of aquatic environments. A prominent example of those is the consistent association of diatoms with *Alphaproteobacteria* of the order *Rhodobacterales*. These photoheterotrophic bacteria have traditionally been described as generalists that scavenge dissolved organic matter. Many observations suggest that members of this clade are specialized in colonizing the microenvironment of diatom cells, known as the phycosphere. However, the molecular mechanisms that differentiate *Rhodobacterales* generalists and phycosphere colonizers are poorly understood. We investigated *Rhodobacterales* in the North Sea during the 2010–2012 spring blooms using a time series of 38 deeply sequenced metagenomes and 10 metaproteomes collected throughout these events. *Rhodobacterales* metagenome assembled genomes (MAGs) were recurrently abundant. They exhibited the highest gene enrichment and protein expression of small-molecule transporters, such as monosaccharides, thiamine and polyamine transporters, and anaplerotic pathways, such as ethylmalonyl and propanoyl-CoA metabolic pathways, all suggestive of a generalist lifestyle. Metaproteomes indicated that the species represented by these MAGs were the dominant suppliers of vitamin B_12_ during the blooms, concomitant with a significant enrichment of genes related to vitamin B_12_ biosynthesis suggestive of association with diatom phycospheres. A closer examination of putative generalists and colonizers showed that putative generalists had persistently higher relative abundance throughout the blooms and thus produced more than 80% of *Rhodobacterales* transport proteins, suggesting rapid growth. In contrast, putative phycosphere colonizers exhibited large fluctuation in relative abundance across the different blooms and correlated strongly with particular diatom species that were dominant during the blooms each year. The defining feature of putative phycosphere colonizers is the presence of the tight adherence (*tad*) gene cluster, which is responsible for the assembly of adhesive pili that presumably enable attachment to diatom hosts. In addition, putative phycosphere colonizers possessed higher prevalence of secondary metabolite biosynthetic gene clusters, particularly homoserine lactones, which can regulate bacterial attachment through quorum sensing. Altogether, these findings suggest that while many members of *Rhodobacterales* are competitive during diatom blooms, only a subset form close associations with diatoms by colonizing their phycospheres.

## Introduction

Marine phytoplankton blooms are an essential part of ocean and global nutrient cycles. These blooms are transient, seasonal and localized events characterized by an increased abundance of algal cells and a spike in the assimilation of CO_2_ and inorganic nutrients (nitrogen and phosphorus) (Behrenfeld et al., 2005; Behrenfeld et al., 2006). This ecosystem imbalance is accompanied by an increase in abundance and activity of heterotrophic bacteria (Buchan et al., 2014). These bacteria consume dissolved and particulate organic matter (DOM/POM) derived from both living and senescent/dead phytoplankton cells (Smith et al., 1992). Both DOM and POM consumption by bacteria contribute significantly to the biogeochemical cycles of carbon, nitrogen, and silicon (Azam and Long, 2001), and lead to remineralization of organic matter back to inorganic constituents required for phytoplankton growth (Bidle et al., 2002).

Helgoland Roads, a long-term ecological research station located in the German Bight of the North Sea hosts annual spring and summer blooms. Spring phytoplankton blooms in these shallow temperate waters are often dominated by diatoms (Eisma, 1987; Giebel et al., 2011), with multiple studies reporting on the composition of bacterioplankton communities (Eilers et al., 2000, 2001; Zubkov et al., 2001; Gerdts et al., 2004; Alderkamp et al., 2006; Rink et al., 2007). During the 2010–2012 spring algal blooms, members of the family *Flavobacteriaceae* were observed to be the most abundant and recurrent bacterial clade (Teeling et al., 2016). Due to their ability to degrade algal polysaccharides, they have thus been a primary focus of research (Xing et al., 2015; Francis et al., 2019, 2021; Kappelmann et al., 2019; Krüger et al., 2019; Avcı et al., 2020). However, members of the order *Rhodobacterales* (also referred to as Roseobacter(s) hereafter), are also frequently found in the North Sea with varying relative abundance (Alderkamp et al., 2006), and have been shown to exhibit significant changes in relative abundance in response to changes in phytoplankton composition and throughout different stages of algal blooms (Rink et al., 2007; González and Moran, 1997; Zubkov et al., 2001). They have also been regularly isolated from phytoplankton cultures (Behringer et al., 2018; Johansson et al., 2019; Wang et al., 2019), indicating that these bacteria are likely to interact closely with phytoplankton. Additionally, members of the Roseobacter group have been shown to influence the microbial community surrounding phytoplankton (Majzoub et al., 2019) and modify growth dynamics of the host (Bolch et al., 2017).

Phytoplankton are expected to be dependent on marine heterotrophic bacteria for supply of various cofactors, while bacteria require phytoplankton photosynthate for growth (Amin et al., 2012; Seymour et al., 2017). Vitamin exchange is among the most important metabolite exchanges that underpin algal-bacterial interactions. Estimates indicate that over half of marine algal species require exogenous sources of vitamin B_12_ (Croft et al., 2005), with the vitamin or its precursors acquired from bacteria, likely in exchange for dissolved organic carbon (Wienhausen et al., 2017). Cooper et al. (2019) demonstrated that cobalamin and thiamine auxotrophy in phytoplankton is alleviated by co-culture with *Dinoroseobacter shibae*, a member of the order *Rhodobacterales*, which in turn receives niacin (B_3_), folate (B_9_) precursors and biotin from the phytoplankton. Aside from vitamin exchange, it has also been demonstrated that the Roseobacter *Sulfitobacter pseudonitzschiae* SA11 (previously *Sulfitobacter* sp. SA11) promotes diatom cell division by the secretion of the hormone indol-3-acetic acid (Amin et al., 2015), which presumably increases DOM output by the diatom. This bacterium also upregulates genes responsible for uptake and assimilation of taurine produced by the diatom.

The region around phytoplankton cells is known as the phycosphere, and molecules within it (at a distance of approximately <100 μm) are mostly transported through diffusion rather than advection (Guasto et al., 2012). The phycosphere is enriched in exuded DOM comprising carbohydrates, amino acids, sugar alcohols and organic acids, which likely serve as chemoattractants for bacteria (Seymour et al., 2010). Recruiting chemotactic bacteria is potentially an important mechanism to establish associations between phytoplankton and beneficial bacteria and recent research suggests chemotaxis plays an important role in the microbial composition of the phycosphere (Raina et al., 2019). Chemotactic bacteria may remain in the phycosphere by attaching to phytoplankton cells or to phytoplankton transparent exopolymeric particles (TEP) (Samo et al., 2018), with attachment resulting in increased nutrient incorporation for both bacteria and phytoplankton and may be a long-term adaptation of specific bacteria ‘symbiotic’ with phytoplankton (Arandia-Gorostidi et al., 2017). Roseobacters have been shown to be the most transcriptionally active taxa in the microbiome of a model diatom, and were able to switch between motile and sessile lifestyles in response to diatom-derived metabolites (Fei et al., 2020; Shibl et al., 2020).

Given the frequency with which Roseobacters are observed to co-occur with phytoplankton, together with their metabolic variability, we sought to differentiate niches of Roseobacters that respond to diatom bloom events through the lens of metagenomics and metaproteomics. Although several members of this group have traditionally been described as ecological generalists, many observations suggest others are specialized in colonizing diatom phycospheres (Newton et al., 2010). However, the molecular mechanisms that differentiate *Rhodobacterales* generalists and phycosphere colonizers are poorly understood. Here, we investigate the interactions of Roseobacters during spring diatom blooms in the North Sea and tease apart the molecular characteristics that distinguish putative generalists from phycosphere colonizers.

## Materials and Methods

### Sampling

Helgoland Roads is a long-term research site that hosts annual spring phytoplankton blooms. Phytoplankton data was assessed on a weekly basis as part of the Helgoland Roads LTER time series and details of data acquisition have been described previously (Teeling et al., 2012; Teeling et al., 2016) (see Supplementary Table 1 for dates on which samples were taken). Samples of bacterioplankton (bacteria and archaea) were collected at the ‘Kabeltonne’ station (54° 11.3′ N, 7° 54.0′ E) near Helgoland island during the 2010, 2011, and 2012 spring algal blooms at bi-weekly to weekly intervals as described previously (Teeling et al., 2012; Teeling et al., 2016). Briefly, surface seawater collected at a depth of ∼1 m was pre-filtered through 10 μm sized filters, followed by 3 μm filtration and finally bacterioplankton were collected on 0.2 μm filters from which DNA was extracted. From this 0.2 to 3 μm size fraction, DNA was extracted for metagenome sequencing and proteins were extracted for metaproteomics (Teeling et al., 2012, 2016).

### Metagenome Assembled Genomes (MAGs)

Thirty-eight surface seawater samples were subject to metagenome sequencing at the DOE Joint Genome Institute on the Illumina HiSeq2000 platform as described previously (Teeling et al., 2016). Quality filtering, raw read trimming, metagenome assembly, binning and MAG refinement was performed through the Anvi’o pipeline as described previously (Krüger et al., 2019). Metagenomes were assembled and binned independently, hence redundant MAGs were obtained. The MAGs were clustered at 95% average nucleotide identity (ANI) which yielded 492 approximate species clusters (hereafter referred to as representative MAGs). Taxonomy of representative MAGs was determined by GTDB-Tk v0.8 (GTDB v89) classification and subsequently deposited into the European Nucleotide Archive under accession PRJEB28156. Representative MAGs with >70% completion or >69% for *Rhodobacterales* MAGs were selected for further analysis (392 MAGs in total). The threshold of completion for *Rhodobacterales* MAGs was lowered to conservatively maximize the number of *Rhodobacterales* MAGs included in downstream analysis (at >70% completion there were 32 *Rhodobacterales* MAGs and at >69% there were 33 MAGs). Metagenome raw reads were mapped to these MAGs at 97% identity. Read counts were normalized against MAG length to obtain reads per kilo base per million mapped reads (RPKM) [number of reads mapped ÷ (length of the MAG in kilobase pairs × total number of mapped reads in dataset ÷ 1,000,000)] and the relative abundance of each MAG in a metagenome was calculated (RPKM_(MAG)_ ÷ RPKM_(__*Sum*__ of *MAGs in metagenome*__)_) as a reflection of *in situ* MAG abundance. MAG relative abundance figures and heatmaps were visualized with R 3.6.1 using ggplot2.

### Metaproteomes

A total of 10 metaproteome from the 2010–2012 Helgoland spring blooms roughly corresponding to pre, middle and post bloom based on chlorophyll *a* concentrations (Supplementary Table 1) were obtained as described in Teeling et al. (2012) and Kappelmann et al. (2019). Briefly, proteins were extracted from bacterioplankton biomass, separated, subject to tryptic digestion and fragment detection carried out using an LTQ Orbitrap Velos mass spectrometer. Spectrometric data is available at the PRIDE repository (Vizcaíno et al., 2016) with the project ID PXD008238. The mass spectrometric data was analyzed using Sequest v27r11 and searches were carried out against a database of proteins from all corresponding metagenomes using USEARCH as described in Krüger et al. (2019). Protein and peptide validation were carried out and normalized spectral abundance factors (%NSAF) were calculated for semiquantitative analysis as previously described (Teeling et al., 2012; Krüger et al., 2019).

### Bioinformatic Analyses

#### Representative MAG Gene Enrichment

Representative MAGs were annotated using EnrichM (v0.5.0) to obtain KEGG Orthology (KO) annotations as described in Boyd et al. (2019)^1^. The *classify* and *enrichment* subcommands were used to determine KEGG module completion and enriched KEGG modules, respectively, among the top four most abundant orders (comparisons between *Rhodobacterales* and *Flavobacteriales*, *Pelagibacterales* and *Pseudomonadales*). The output files for presence/absence of KOs determined by Fisher’s exact test and overrepresentation within a group determined by Mann-Whitney U test were further refined based on significance (corrected *p* value <0.05) and presence of the KO in >50% of MAGs within an order. KO annotations of significance were searched against the KO database^2^ (Kanehisa et al., 2016) to obtain the number of enriched KOs that belong to specific BRITE hierarchies. The resulting heatmap was generated with GraphPad Prism 9.

#### Metaproteomic Analysis

Using standalone BLAST v2.10.0 +, predicted protein sequences from representative MAGs (392) were queried against a redundant metaproteome sequence database (3,212,324 sequences). Hits were filtered for percent identity >99% and *e*-value <1e-10. Proteins having significant hits to multiple MAGs were excluded from downstream analysis to avoid incorrectly assigning a protein to a MAG which may skew relative abundance calculations. The proteins with definitive hits against MAGs were queried against the KEGG database using an implementation of KofamKOALA to obtain KO numbers (Aramaki et al., 2020). BRITE hierarchical categories of the KO numbers were obtained with R package KEGGREST 1.24.1. The top 10 most abundant protein categories in the 2010 – 2012 metaproteome were identified based on the total number of unique proteins per BRITE category. The %NSAF of proteins from each category were summed and the log of relative abundance was calculated for individual years and phase of bloom for each of the four most abundant taxonomic groups. The data plot was created and visualized with RStudio (Version 1.3.1093) using ggplot2.

#### Phylogenomics of *Rhodobacterales* MAGs

The *Rhodobacterales* MAGs were phylogenetically placed among 62 complete or draft genomes from the *Rhodobacteraceae* family downloaded from NCBI (Supplementary Table 2). Functional annotation of the representative MAGs and publicly available Roseobacter group genomes was performed using Prokka v1.14.0. Concatenated sequences of 107 single-copy core genes were obtained using bcgTree (Ankenbrand and Keller, 2016) implemented with HMMER v3.1b2 (Eddy, 2009), MUSCLE v3.8.31 (Edgar, 2004) and Gblocks 0.91b (Castresana, 2000) to create and refine a multiple sequence alignment. The phylogenomic tree was generated using RAxML (Stamatakis, 2014) through the ETE3 Python package (Huerta-Cepas et al., 2016) with predefined workflow standard_trimmed_raxml_bootstrap. The unrooted tree was visualized on the Interactive Tree of Life (iTOL) v5 (Letunic and Bork, 2019).

#### Niche Differentiation of *Rhodobacterales* MAGs

Significant differences in MAG size among *Rhodobacterales* phylogenetic groupings were confirmed by Student’s *t* test (*p* value <0.05). All *Rhodobacterales* MAGs were submitted to secondary metabolite gene cluster analysis using ARTS 2.0 (Mungan et al., 2020), which implements antiSMASH 5.0 (Blin et al., 2019). Presence of biosynthetic gene clusters (BGCs) was tabulated. Given the inability to effectively differentiate *Rhodobacterales* based solely on phylogeny and secondary metabolism, the MAGs were reorganized into two groups based on their correlation to dominant diatom species during the blooms. MAG relative abundances were centered log-ratio transformed prior to statistical analysis. Redundancy analysis (RDA) was first used to infer the underlaying relationship between diatoms and Roseobacter MAGs using the R package, *vegan* (Version 2.5-7) (Oksanen et al., 2020). RDA is a constrained multivariate ordination technique that extracts gradients of variation in dependent variables (Roseobacter MAG relative abundance) explainable by independent variables (diatom cell counts) and assumes a linear relationship. Diatom cell counts were normalized by Hellinger-transformation prior to RDA to ensure the data met the statistical assumption of linearity. The significance of variation in Roseobacter MAG abundances explained by the explanatory variables was tested using an ANOVA-like Monte Carlo permutation test as implemented in *vegan*. Following confirmation of the relationship between Roseobacter MAGs and diatom species, diatom cell counts and MAG relative abundance were used to calculate Spearman rank correlation coefficients using GraphPad Prism 9. MAGs with statistically significant (*p* value <0.05) positive correlation coefficients, indicative of positive relationships to specific diatom species, were identified. The relationships between MAGs and diatoms with positive correlations was visualized with Cytoscape v3.8.2. MAGs with no significant correlations to diatoms, those that were significantly negatively correlated to diatoms and those MAGs that had consistently high relative abundance were not considered as potential phycosphere colonizers. Consequently, a subset of MAGs with positive correlations to diatoms were categorized as potential phycosphere colonizers while the remaining *Rhodobacterales* MAGs were categorized as putative generalists. The *Rhodobacterales* MAGs were analyzed using Anvi’o 6.1 (Eren et al., 2015) following the microbial pangenomics workflow^3^ in order to identify core, and enriched genes and functions based on the original phylogenetic groups and importantly, niche groupings.

#### Taxonomic Distribution and Completeness of the *Tad* Gene Locus

The *Aggregatibacter actinomycetemcomitans* TadA protein sequence (NCBI Accession No. AF152598) was used to search for homologs with BLASTX against the non-redundant protein sequences (nr) database and the resulting top 100 hits with percent identity > 60% and *e*-value = 0.0 were downloaded. These 100 TadA homologs together with TadA homologs from the *Rhodobacterales* MAGs were aligned with an online implementation of MUSCLE (Madeira et al., 2019) and the resulting multiple sequence alignment in ClustalW format used to generate a hidden Markov model (HMM) profile on HMMER v3.2.1 with hmmbuild. The hmm profile was queried against the Tara Oceans Microbiome Reference Gene Catalog version 1 on the Ocean Gene Atlas^4^ webserver (Villar et al., 2018) with an e-value threshold of 1e-50. Taxonomic abundances of homologs found in surface and deep chlorophyll maximum samples across all size fractions (0–3 μm) and geographical locations was visualized as a single donut plot.

The *Rhodobacterales* MAGs, *A. actinomycetemcomitans* (available on RAST), *Sulfitobacter pseudonitzschiae* F5, *Phaeobacter* sp. F10 and *Alteromonas macleodii* F12 genomes (GenBank accession numbers WKFG01000000 and CP046140-CP046144 reported in Fei et al. (2020) were annotated using RAST (Aziz et al., 2008) in order to identify all genes and homologs that constitute the *tad* gene locus. Presence and completion of the *tad* gene cluster, also known as the Widespread Colonization Island (WCI) was determined by comparison against the *A. actinomycetemcomitans tad* cluster on SEED Viewer 2.0 (Overbeek et al., 2014). GBK files were downloaded and viewed in Geneious 11.1.5 and regions containing *tad* related genes were extracted and visualized with RStudio (Version 1.3.1093) using gggenes^5^.

## Data Availability Statement

The metagenomic dataset PRJEB28156 for this study can be found in the European Nucleotide Archive under accession [https://www.ebi.ac.uk/ena/data/view/PRJEB28156]. The metaproteomic spectrometric data ID PXD008238 for this study can be found in the PRIDE repository (Vizcaíno et al., 2016).

## Results and Discussion

### MAG *in situ* Abundances and Comparative Genomics

We obtained 492 representative MAGs from 38 previously assembled 2010-2012 Helgoland metagenomes, of which 392 MAGs with CheckM completion > 69% for *Rhodobacterales* and > 70% for all other taxa were selected for downstream analysis (Supplementary Table 3). The abundances of these MAGs in the bloom samples were assessed by metagenomic read recruitment. These representative MAGs showed a pattern of succession across the three consecutive years that corroborated 16S rRNA gene amplicon sequencing data published by Teeling et al. (2016), which reported increased relative abundance of *Bacteroidetes* at the onset of the bloom, followed by *Gammaproteobacteria* and subsequently surpassed by *Alphaproteobacteria*. Similar patterns of abundance and recurrence have been reported in response to phytoplankton blooms (Williams et al., 2013; Needham and Fuhrman, 2016; Zhang et al., 2020) with heterotrophic members of the *Bacteroidetes*, *Gammaproteobacteria* and *Alphaproteobacteria*, particularly, *Rhodobacterales*, being among those groups that respond to phytoplankton-derived polysaccharides (Buchan et al., 2014). At a finer level of taxonomic resolution, it was revealed that *Rhodobacterales* MAGs were among the most recurrent and abundant throughout the datasets, in addition to *Flavobacteriales*, *Pelagibacterales* and *Pseudomonadales* (Supplementary Figure 1). *Flavobacteriales* are typically first responders to phytoplankton blooms consistent with their ability to break down complex algal derived organic matter, making labile compounds available to alphaproteobacterial populations that are generally dominated by the SAR11 clade and *Rhodobacterales* (Teeling et al., 2012; Williams et al., 2013).

Investigation into the transporter gene profile and expression of these transports suggest a difference in nutritional strategies along taxonomic lines. During the North Sea spring phytoplankton bloom of 2009, Teeling et al. (2012) showed that *Gammaproteobacteria* and *Flavobacteria* display similar transporter expression profiles with a dominant expression of TonB-dependent transporter (TBDT) components that are known to facilitate uptake of large compounds such as oligosaccharides, siderophores and vitamin B_12_. On the other hand, *Alphaproteobacteria* and particularly, Roseobacters, showed high expression levels of adenosine triphosphate (ATP)–binding cassette (ABC) and tripartite ATP-independent periplasmic (TRAP) transporters for low-molecular-weight substrates. A subsequent metagenomic survey of four consecutive North Sea spring blooms (2009 – 2012) confirmed that TBDT genes were prevalent in *Bacteroidetes* while TRAP transporters were mostly present in *Alphaproteobacteria* (Teeling et al., 2016). In light of this and given that *Rhodobacterales, Flavobacteriales*, *Pelagibacterales* and *Pseudomonadales* MAGs were the most abundant taxa (Supplementary Figure 1), we investigated the metabolic differences between these four major orders by searching for enriched KEGG orthologies (KO) and modules within the respective groups. With the relatively low number of *Rhodobacterales* MAGs (33 MAGs), we thought it prudent to compare the *Rhodobacterales* MAGs against each order individually rather than comparing them against the other orders collectively (359 MAGs), thus reducing the risk of diluting potential nuanced differences. In all scenarios *Rhodobacterales* were enriched for a number of KOs over diverse protein categories (EnrichM analysis) (Supplementary Figure 2 and Supplementary File 1). *Pelagibacterales*, like *Rhodobacterales*, is classified as an alphaproteobacterial order, however there still appears to be distinct KO enrichment in the latter, suggesting a more diverse metabolic potential when compared to other *Alphaproteobacteria*. Hogle et al., 2016 reported that *Rhodobacterales* have a more versatile array of trace metal (e.g., nickel, zinc, manganese, and iron) uptake transporters when compared to SAR11 (*Pelagibacterales*), which have more streamlined genomes. In the current study it is observed that *Rhodobacterales* are enriched for nickel, molybdate and tungstate transporters when compared to *Pelagibacterales* (Supplementary File 1). *Rhodobacterales* possess enriched KOs for peptidases and inhibitors, amino acid related proteins, DNA repair and recombination proteins, ribosome biogenesis, tRNA biogenesis, secretion system and membrane trafficking compared to *Pelagibacterales*. Similar categories are enriched in *Rhodobacterales* MAGs when compared to *Flavobacteriales* and *Pseudomonadales*, however, the enrichment profile is much more pronounced. Other categories that are enriched in *Rhodobacterales* when compared to *Flavobacteriales* and *Pseudomonadales* include two-component systems, lipid biosynthesis proteins, lipopolysaccharide biosynthesis proteins and photosynthesis proteins. Enriched KOs in *Rhodobacterales* among all comparisons, of particular note, include those related to transport proteins (confirming the metagenomic survey in Teeling et al., 2016) and porphyrin and chlorophyll metabolism.

Transport proteins were the most highly enriched category in *Rhodobacterales* MAGs and porphyrin and chlorophyll metabolism is of interest as vitamin B_12_ biosynthetic intermediates are produced through this metabolic network. Specific KEGG modules that were consistently enriched in *Rhodobacterales* relative to the other orders were transporters of monosaccharides, thiamine and polyamines (Supplementary File 1). Physiological studies have shown that Roseobacters are able to grow on a wide variety of substrates and this versatility is reflected in their high number of transport proteins (Giebel et al., 2013; Voget et al., 2015). Anaplerotic pathways, including ethylmalonyl and propanoyl-CoA metabolic pathways were also enriched in *Rhodobacterales* MAGs. These pathways offer alternate metabolic routes for carbon assimilation. They promote growth on precursor metabolites (intermediates of central carbon metabolism) that replenish tricarboxylic acid (TCA) cycle intermediates, thus feeding central metabolism (Carlson et al., 2019). Collectively, these functional and metabolic features are suggestive of a generalist lifestyle commonly associated with members of the *Rhodobacterales*.

Additionally, enzymes related to cobalamin (vitamin B_12_) biosynthesis and a tricarboxylic acid transport two-component regulatory system (TctD-TctE) were enriched in the *Rhodobacterales* MAGs. All but one of the *Rhodobacterales* MAGs possessed a complete biosynthetic pathway for vitamin B_12_. *Rhodobacterales* MAG 20160419_Bin_37_1 lacked most of the biosynthetic pathway but does still possess the final enzyme in the pathway to produce vitamin B_12_ coenzyme. This confirms findings that the majority of Roseobacters have evidence of functioning cobalamin biosynthesis pathways, in contrast to more than 60% of marine bacterial species that are unable to synthesize vitamin B_12_ (Luo and Moran, 2014; Sanudo-Wilhelmy et al., 2014). Phytoplankton are often auxotrophic (∼50% of >300 surveyed phytoplankton) for vitamin B_12_; the ability of Roseobacters to produce vitamin B_12_ and their co-occurrence with phytoplankton may be indicative of interactions between them. The TctD-TctE system plays a role in resistance and tolerance to aminoglycosides in *Pseudomonas aeruginosa* within biofilms and thus contributes to persistence of bacteria within these mixed communities (Taylor et al., 2019). These features bring to light an alternate lifestyle of Roseobacters, one that is suggestive of association with phytoplankton phycospheres.

### Metaproteomics

**FIGURE 1:**
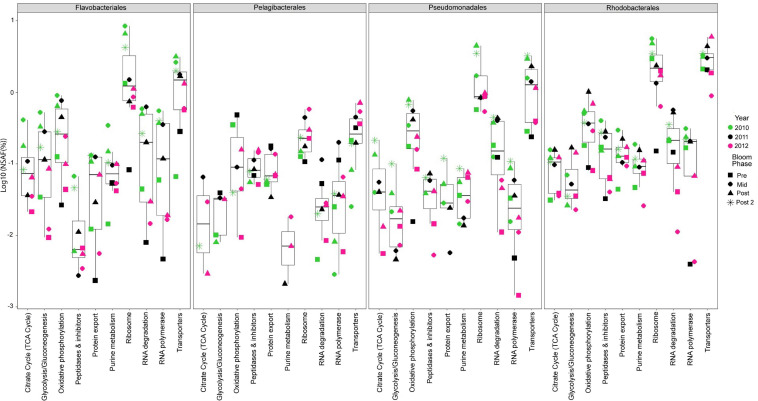
Top ten most abundant protein categories in the 2010 – 2012 metaproteomes. Each point represents the log of the relative abundances of all the proteins (cumulative%NSAF) that belong to a particular category during a specific year (symbol color) and phase of the bloom (symbol shape) and among the four most abundant taxonomic groups. Bloom phases were determined based on *chl a* concentration as reported in Teeling et al. (2016) (Supplementary Table 1). The box plots provide descriptive statistics of all datapoints for a specific category.

We analyzed 10 metaproteomes from the 2010 to 2012 spring algal blooms to investigate the *in situ* expression profiles of MAGs from the four most abundant orders and whether protein expression correlates with gene enrichment analyses. The proteins were compared via BLAST against all 392 representative MAGs and significant protein hits were subsequently placed into functional categories based on BRITE hierarchies. There were 5175 proteins that mapped to the four most abundant orders (Supplementary Table 4) with 66.9% of these proteins having KO annotations and accounting for 47% of all proteins that were mapped to representative MAGs. The 10 most abundant protein categories throughout the entire metaproteomics dataset and across all four abundant orders were related to central metabolism and cellular processes such as proteins found in glycolysis, the TCA cycle, ribosomes and RNA polymerases (Figure 1). In each category and in each of the four most abundant order there was a noticeable increase in protein relative abundance (%NSAF) as blooms progressed each year. Salazar et al., 2019 reported that community turn over and changes in gene expression could influence transcript abundance. At first glance the protein expression profile of the four most abundant orders appears to increase with their relative abundance as the bloom progresses (Figure 1 and Supplementary Figure 1), suggesting gene copy number may be responsible for the perceived change in protein expression; however, there are specific instances, such as mid/post-bloom expression of transport proteins (2010, 2011 and 2012), where even at lower relative abundance than other orders, *Rhodobacterales* display higher levels of relative protein expression.

Consistent with the EnrichM KO enrichment analysis, *Rhodobacterales* MAGs consistently expressed the highest relative abundance (%NSAF) of transport proteins. Most of these transport proteins were annotated as amino acid transporters, low molecular weight sugar transporters (xylose, sorbitol/mannitol, fructose), putrescine transporters and microcin C transporters (Supplementary Table 4). Primary production due to phytoplankton blooms are assumed to be responsible for the availability of dissolved free carbohydrates such as glucose and other monosaccharides. It has been reported that Roseobacters were highly active during spring phytoplankton blooms in the North Sea and largely responsible for the uptake of glucose (Ittekkot et al., 1981; Alonso and Pernthaler, 2006).

**FIGURE 2:**
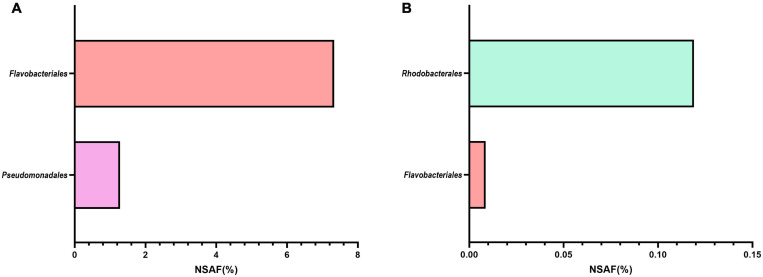
Metaproteome expression of **(A)** cobalamin transport and **(B)** biosynthesis proteins produced by MAGs belonging to the four most abundant orders during blooms across 2010-2012. NSAF (%) represents the summed percent normalized spectral abundance factor of transport or biosynthesis proteins. Cobalamin transport and biosynthesis proteins from *Pelagibacterales* were not detected. The number of transport proteins contributing to the total NSAF(%) for the respective taxonomic group are as follow: *Flavobacteriales*: 76; *Pseudomonadales*: 23. *Rhodobacterales* cobalamin transport proteins were not detected. The number of cobalamin biosynthesis proteins contributing to the total NSAF(%) for the respective taxonomic group are as follow: *Flavobacteriales*: 1; *Rhodobacterales*: 4. Only the last enzyme in the cobalamin biosynthetic pathway was taken into consideration for this calculation: MMAB/pdu = Cobalamin adenosyltransferase; cobW = cobalamin biosynthesis protein.

Additionally, we looked specifically for the presence of proteins related to cobalamin transport and biosynthesis (Figures 2A,B). Representative MAGs of *Flavobacteriales* and *Pseudomonadales*, but not *Pelagibacterales* or *Rhodobacterales*, only expressed a number of cobalamin receptor proteins (Figure 2A). These receptor proteins bind extracellular vitamin B_12_ with high affinity before subsequent uptake (Pienko and Trylska, 2020). Confirming EnrichM gene enrichment analysis, proteins involved in the biosynthesis of vitamin B_12_ were produced almost exclusively by *Rhodobacterales* MAGs (Figure 2B). Only five proteins related to the direct production of vitamin B_12_ were found in the metaproteome, with only one produced by a *Flavobacteriales* MAG and expressed solely during the 2010 bloom while the remaining four proteins were produced by *Rhodobacterales* MAGs across multiple blooms. These findings corroborate the enrichment analysis that Roseobacters are likely the dominant suppliers of vitamin B_12_ during these blooms. Vitamin B_12_ is limiting in the ocean and bacteria in surface waters will likely compete with algae for vitamin B_12_ (Grant et al., 2014; Sanudo-Wilhelmy et al., 2014). Many phytoplankton-bacteria interactions hinge on the exchange of B vitamins and studies have shown that phytoplankton species meet their vitamin B_12_ requirement by entering into symbiotic relationships with specific bacteria, often times with Roseobacters, that provide vitamin B_12_ in exchange for fixed carbon and other metabolites (Croft et al., 2005; Grant et al., 2014; Durham et al., 2015; Cooper et al., 2019). In order to minimize competitive vitamin B_12_ uptake by *Flavobacteriales* and *Pseudomonadales* MAGs, the exchange of metabolites between *Rhodobacterales* MAGs and phytoplankton is likely to occur within the phycosphere, which may increase uptake of the vitamin by phytoplankton relative to vitamin production by free-living bacteria. Thus, we sought to identify specific *Rhodobacterales* MAGs that interact directly with diatoms by colonizing the phycosphere.

### Niche Differentiation of *Rhodobacterales* MAGs

**FIGURE 3:**
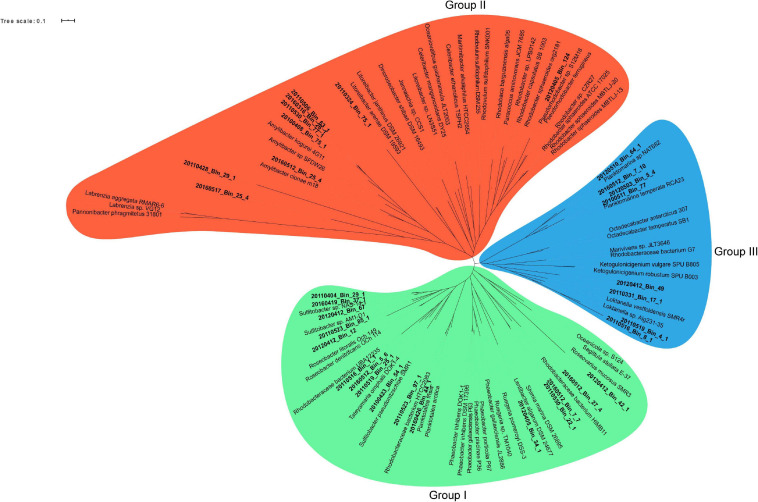
Maximum likelihood unrooted phylogenomic tree of 107 conserved single copy genes from spring bloom associated *Rhodobacterales* MAGs (bold-faced) and publicly available *Rhodobacterales* genomes. Three branch points were identified and served as the basis for grouping the MAGs and genomes. Group I: Green, Group II: Red and Group III: Blue. The maximum likelihood phylogenomic tree was generated using RAxML and bootstrapping was performed at 100 iteration. Bootstrap values and sequence alignment can be found in the raw RAxML output file (Supplementary File 2).

A phylogenetic survey of the 33 *Rhodobacterales* MAGs together with 62 publicly available *Rhodobacterales* genomes based on 107 conserved single copy genes (Ankenbrand and Keller, 2016) revealed that the MAGs fell into three distinct groups (Figure 3). There were 16, 9 and 8 MAGs that belonged to Groups I, II and III, respectively. Some of these MAGs were closely related to Roseobacters that were typically found in high abundance in North Sea waters, such as *Planktomarina temperata* RCA23 (20120510_Bin_64_1; 20160512_Bin_7_10; 20120503_Bin_5_4; 20100511_Bin_77) (Giebel et al., 2013) and *Planktotalea frisia* (20160426_Bin_44_1; 20110526_Bin_97_1) (Hahnke et al., 2012). Other MAGs were closely related to *Amylibacter* (20160512_Bin_25_4; 20100408_Bin_75_1; 20110530_Bin_77_1; 20160316_Bin_26_1; 20110506_Bin_63_1) and *Sulfitobacter* species (20110404_Bin_29_1; 20160419_Bin_37_1; 20160412_Bin_67; 20110523_Bin_85_1; 20120412_Bin_12; 20100423_Bin_54_1). Members of the Roseobacter group are highly diverse and span over 70 genera and hundreds of species (Pujalte et al., 2014; Simon et al., 2017). Most members of the Roseobacters have large genomes and display a high degree of genome plasticity that affords the Roseobacters their adaptability and diversity, while other pelagic Roseobacters have streamlined genomes and are possibly adapted to oligotrophic lifestyles (Luo and Moran, 2014; Luo et al., 2014; Voget et al., 2015; Simon et al., 2017). The MAGs in Group I (3.32 Mb ± 0.61 Mb) were found to have larger genomes than MAGs in Groups II (2.57 Mb ± 0.69 Mb) and III (2.71 Mb ± 0.31 Mb) (*t*-test, *p* value < 0.01 and < 0.05, respectively) (Supplementary Figure 3), suggesting a more diverse metabolic potential. Konstantinidis and Tiedje (2004) reported that larger genomes were found to be disproportionately enriched in genes related to secondary metabolism. Therefore, we postulated that the success of specific *Rhodobacterales* MAGs in colonizing diatom phycospheres might be linked to a suite of unique secondary metabolites that may enable or enhance phycosphere colonization. The MAGs were thus interrogated for the presence of potential biosynthetic gene clusters related to secondary metabolism (Table 1).

**TABLE 1:** Putative biosynthetic gene clusters required for the production of secondary metabolites in *Rhodobacterales* MAGs.

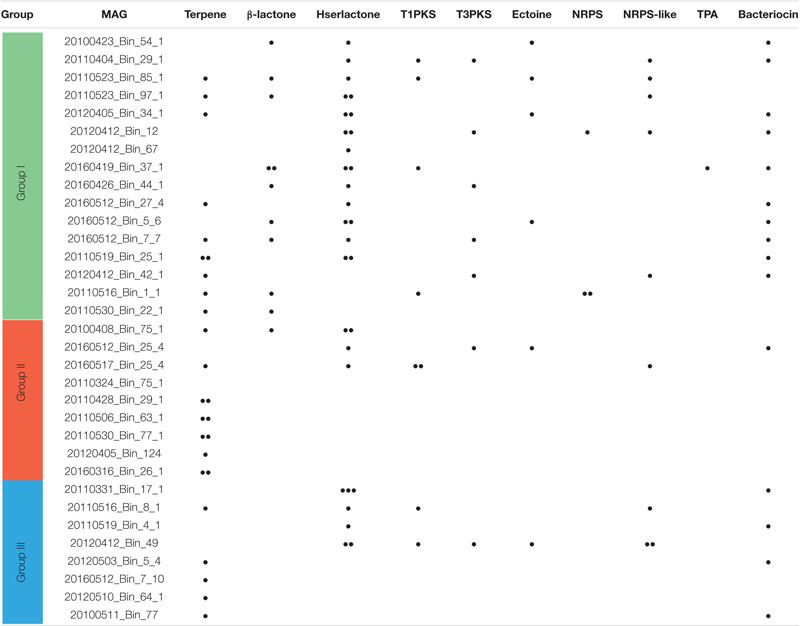

*Each • symbol represents a single complete biosynthetic gene cluster, hence • • or • • • means two or three complete gene clusters, respectively. Hserlactone: Homoserine lactone; T1PKS: Type I Polyketide synthase, T3PKS: Type III Polyketide synthase; NRPS: Non-ribosomal Peptide Synthase; TPA: Tropodithietic-acid.*

Group I MAGs had a more diverse genetic repertoire of secondary metabolites with the majority of them presenting with elevated counts of homoserine lactone biosynthetic gene clusters, which are responsible for the production of quorum sensing (QS) molecules. Quorum sensing systems utilize small diffusible molecules to synchronize gene expression of a bacterial population in a density-dependent manner. *N*-acyl-homoserine lactone (AHLs) are the most commonly described QS signaling molecules in *Proteobacteria*, which are synthesized by an autoinducer synthase (LuxI) and bind to an autoinducer regulator (LuxR) to regulate a variety of genes (Case et al., 2008; Cude and Buchan, 2013). QS systems have been described in Roseobacters and have been implicated in the colonization of particulate matter (marine snow) in the ocean (Gram et al., 2002). Roseobacters isolated from diatom microbiomes are able to attach to and colonize diatom cells in response to particular AHL signals they produce (Fei et al., 2020). Group I MAGs also displayed a high prevalence for the production of putative antimicrobial compounds, such as bacteriocins, β-lactones and polyketides. These secondary metabolites have been shown to have potent bioactivity against bacteria and fungi (Ridley et al., 2008; Cotter et al., 2013; Robinson et al., 2019) and may play a role in chemical warfare within the phycosphere by eliminating competitors and protecting phytoplankton cells from potential pathogens. Several members from Groups II and III appear to share a similar biosynthetic gene profile and thus the identification of potential colonizers cannot be based solely on phylogeny. Indeed, Newton et al. (2010) reported that phylogenetic tree topology was not the best model for organizing genome characteristics in Roseobacters and that it was a lifestyle framework that better suited grouping of Roseobacter isolates. Thus, we organized and grouped the *Rhodobacterales* MAGs by their abundance relative to different diatom species occurring in the different blooms.

**FIGURE 4:**
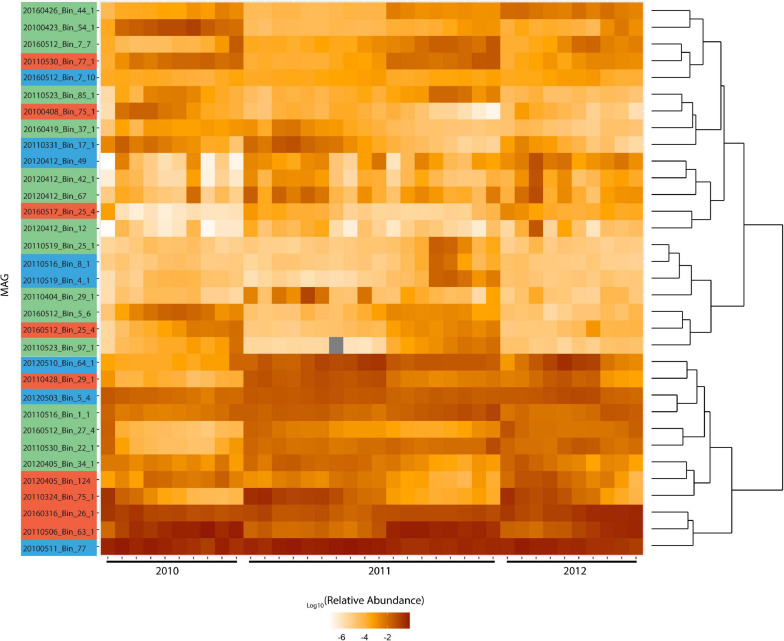
Log relative abundance of *Rhodobacterales* MAGs during blooms across 2010-2012. Color coding of MAG names indicate the MAG grouping based on Figure 3. Metagenome raw reads were mapped to these MAGs at 97% identity. Cells with gray shading indicate values that are zero. Read counts were normalized against MAG length and the relative abundance calculated. Hierarchical clustering was performed in and visualized with R 3.6.1 using ggplot2. Distance method: canberra and hierarchical clustering method: ward.D2 were used. Upper cluster represents MAGs with fluctuating abundance while lower cluster represents MAGs with consistently high abundance throughout blooms.

The MAGs were clustered based on their relative abundance during the blooms, which yielded a distinct bifurcation in their hierarchical clustering (Figure 4). The two clusters represent those that are consistently present in high abundance throughout the blooms and those that show clear patterns of fluctuation within and across different blooms. There was no pattern that indicates broad phylogenetic groupings has any bearing on the response to phytoplankton succession. In marine ecosystems it has been shown that finer levels of taxonomy can display stronger patterns of correlation which can unravel specific interactions, such as those between microbial hosts and their viruses (Needham et al., 2017). Interestingly, Chafee et al. (2018) reported both broad (those that occur throughout the year) and narrow (those that are enriched during spring and summer) recurrent patterns of *Rhodobacterales* in response to seasonal bloom dynamics. Roseobacter group oligotypes of *Amylibacter* and *Planktomarina* were among five broadly recurrent taxa which collectively contributed to ∼20% of the total community throughout the year while oligotypes of *Sulfitobacter*, *Roseobacter* and *Loktanella* among others, were recurrent and enriched during spring and summer when phytoplankton blooms occur (Chafee et al., 2018). These findings further support the distinction between Roseobacter generalists and close algal associates.

**FIGURE 5:**
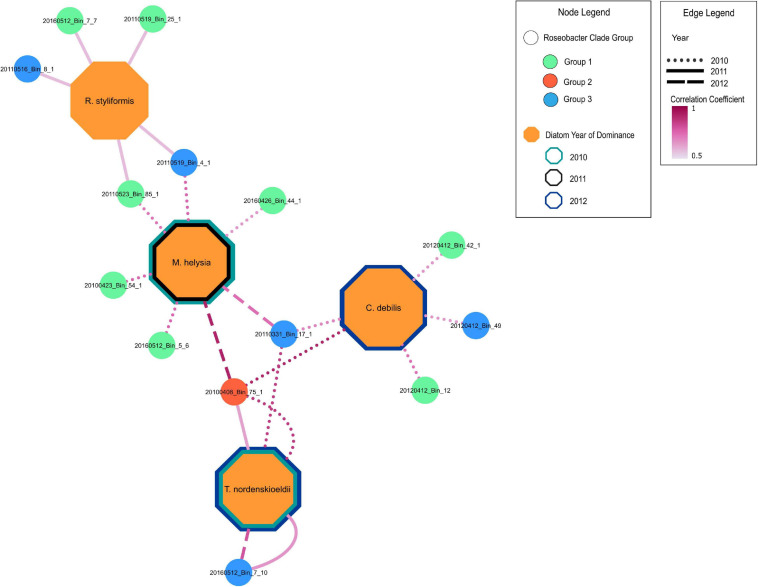
Positive correlation relationships of MAGs with dominant diatom species during blooms across 2010-2012. MAGs nodes are color coded according to groups in Figure 3. Only significant (*p* < 0.05) positive Spearman rank correlation coefficients between MAGs and significant diatoms (from RDA analysis) are shown (Supplementary Table 5). Border color of diatom nodes indicates the year of dominance. Diatoms were considered dominant if they had the highest diatom cell count during bloom peaks. The 2010 bloom was bimodal with *Thalassiosira nordenskioeldii* and *Mediopyxis helsia* peaking at different times. The 2012 phytoplankton bloom was dominated by a silicoflagellate, yet *Thalassiosira nordenskioeldii* and *Chaetoceros debilis* were still present, had very similar cell counts and were thus both considered the dominating diatom species. Edge line type indicates the year in which a positive relationship is present between a MAG and a diatom species. The edge line shading indicates the strength of the correlation.

Redundancy analysis (RDA) was carried out to assess the degree to which the Roseobacter MAGs were associated with different diatom species during the individual blooms (Supplementary Figures 4A-C). This analysis revealed that 69, 37, and 64% (adjusted-R-squared value of the model) of the variation observed in the Roseobacter MAG abundances can be explained by diatom abundance in 2010, 2011 and 2012, respectively. The relationship between Roseobacter abundance and diatoms were significant in 2010 (Monte Carlo Permutation test, *n* = 999; *F* = 6.05; *p* = 0.001) (Supplementary Figure 4A), 2011 (Monte Carlo Permutation test, *n* = 999; *F* = 2.75; *p* = 0.029) (Supplementary Figure 4B) and 2012 (Monte Carlo Permutation test, *n* = 999; *F* = 4.99; *p* = 0.001) (Supplementary Figure 4C). In order to refine the distinction between putative generalists and phycosphere colonizers, MAG relative abundances (Figure 4) and diatom cell counts (Supplementary Figure 5) were used to compute Spearman rank correlation coefficients, which provide information on the strength of the relationship between specific MAGs and diatom species. Twenty-two MAGs had statistically significant (*p* value < 0.05) positive correlation coefficients that were all greater than 0.5, indicating moderate to strong positive relationships to specific diatom species (Supplementary Table 5). MAGs with no significant correlations to diatoms, those that were only negatively correlated to diatoms and those MAGs that had consistently high relative abundance (from Figure 4) were not considered as potential phycosphere colonizers. Consequently, 14 MAGs with positive correlations to diatoms were identified as potential colonizers (Figure 5) which were then compared against the remaining 19 Roseobacter MAGs that were categorized as putative generalists. It should be noted that due to the low number of independent samples (*n* = 3, one per year), these correlations are not as robust as we would like it to be.

### Putative Generalists vs. Phycosphere Colonizers

**FIGURE 6:**
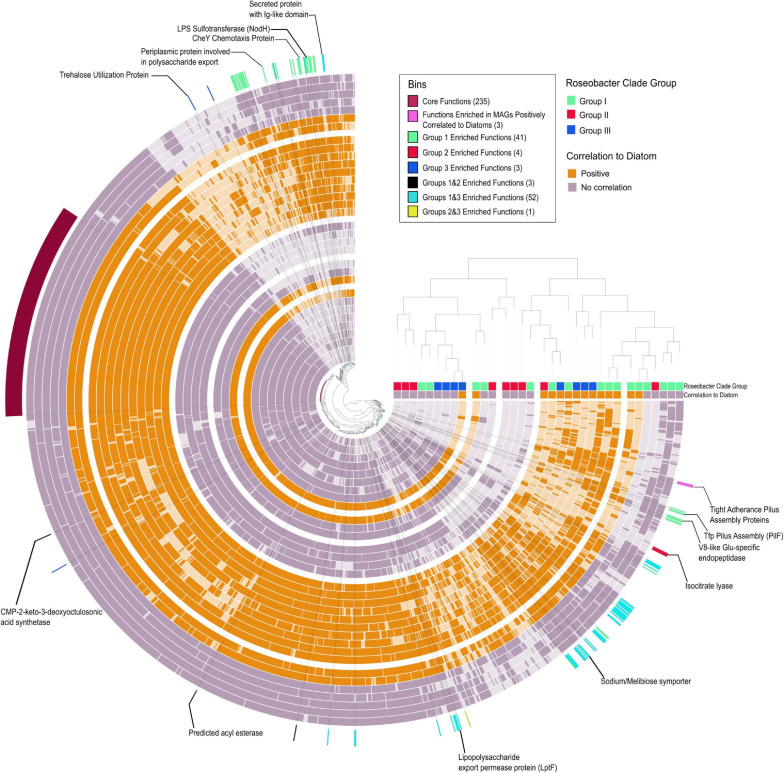
Pangenomic and enrichment analysis of *Rhodobacterales* MAGs. Concentric circles represent individual MAGs with dark shading indicating the presence of a COG and light shading indicating absence of a COG. The central tree is based on COG functional occurrence in a MAG and the tree on the right is based on overall functional similarity between the MAGs. Bins indicate functions (COGs) that are significantly enriched in a specific group of MAGs. Functions enriched in MAGs positively correlated to diatoms selected based on the criteria of being present in >70% putative colonizers and < 20% in putative generalists. Numbers in parenthesis beside the bin name indicated the number of COGs in that bin (Supplementary Table 6). Positively correlated MAGs are considered putative phycosphere colonizers and non-correlated MAGs are considered putative generalists.

There are a number of studies that provide an overview of Roseobacter genome content (Moran et al., 2004; Buchan et al., 2005; Moran et al., 2007; Brinkhoff et al., 2008; Newton et al., 2010; Luo and Moran, 2014), however, metagenomics surveys have demonstrated that our view of the ecology of Roseobacters are skewed and biased toward cultured Roseobacters (Luo et al., 2012; Luo and Moran, 2014). Thus, we attempted to tease apart the genomic differences between bloom associated Roseobacters to understand if only a subpopulation of Roseobacters is able to colonize the phycosphere (Anvi’o enrichment analysis) (Figure 6 and Supplementary Table 6). There were 235 core protein functions [clusters of orthologous groups (COGs)] shared amongst the 33 *Rhodobacterales* MAGs that included general cellular processes, central metabolism and transport proteins that correspond to the enriched transporters discussed earlier. Members of Group I displayed the greatest number of enriched functions (41 enriched COGs), with many of them involved in chemotaxis, biofilm formation and attachment. This suggests that Group I members have a greater potential to successfully colonize the phycosphere. In contrast, Group II was only enriched in 6 COGs, one of which is isocitrate lyase, which is important in the glyoxylate pathway and serves as an alternate to the TCA cycle to enable bacteria to convert fatty acids into carbohydrates (Maharjan et al., 2005). Polyunsaturated fatty acids are produced by a number of microalgae and have a considerable contribution to the marine food web and may serve as a carbon source for marine microbes and eukaryotes (del Rosario González-Baró and Pollero, 1998; Sasso et al., 2012). Group III MAGs displayed 3 enriched functions, one of them being a trehalose utilization protein. Some microalgae are known to produce high amounts of trehalose at night (Hirth et al., 2017), suggesting interactions between Group III members and phytoplankton could be diurnally regulated. Cumulatively, these findings suggest that distinct diatom metabolites may serve as unique currencies to a select few groups of Roseobacters. Although Group I members were significantly enriched for functions associated with interactions with phytoplankton, as observed with secondary metabolism, specific members from Groups II and III possess some, or all these features as well.

Based on the correlation analysis with diatom cell counts (Figures 4, 5), putative generalist and phycosphere colonizer MAGs were investigated for gene enrichments. The enrichment analysis did not identify COGs that were significantly enriched, however, specific COGs which met a strict selection criterion (> 70% occurrence in putative colonizers and < 20% occurrence in generalists) were of particular interest. Surprisingly, three COGs (CpaF, TadB and TadC) that contribute to the production of tight adherence (Tad) pilus proteins were the only features that set putative phycosphere colonizers apart from the putative generalists (Figure 6 and Supplementary Table 6). A potential caveat exists in that the *Rhodobacterales* MAGs are not complete and may influence gene presence/absence and enrichment. However, given that potential phycosphere colonizers are less complete on average (88%) than generalists (91%), there would be bias against phycosphere colonizers and thus, this gives us more confidence in our results. These proteins make up a secretion system (a subtype of type IV secretion systems) that is required for the assembly and secretion of adhesive fimbrial low-molecular-weight proteins, otherwise known as Flp pili (Tomich et al., 2007). Flp pili have been shown to contribute to the attachment of *Pseudomonas aeruginosa* to both biotic and abiotic surfaces (de Bentzmann et al., 2006) and *tad* genes were proven to be essential through transposon mutagenesis for biofilm formation, colonization and pathogenesis in a number of genera in diverse niches (Kachlany et al., 2001; Clock et al., 2008; Pu et al., 2018). In contrast to other type IV pilus systems, Tad clusters seem to lack dedicated retraction machinery and most Tad pili do not seem to be dynamic, precluding twitching motility (Tomich et al., 2007). However, recent findings by Sangermani et al. (2019) show that the *Caulobacter crescentus* Tad pili mediates initial attachment to surfaces and undergoes repeated cycles of extension and retracting which helps reorient cells ahead of adhesive exopolysaccharide (EPS) (holdfast) production which establishes long-term attachment. The presence of adhesive pili and holdfasts have previously been reported in marine Roseobacters (Gonzalez et al., 1997; Slightom and Buchan, 2009; Bartling et al., 2018), yet this is not a feature that is universal to Roseobacters as described by Voget et al. (2015). The prevalence and importance of the *tad* gene locus in Roseobacters in association with diatoms has not been adequately investigated. The *tad* locus was first described in *Aggregatibacter (Actinobacillus) actinomycetemcomitans*. It is composed of 14 genes (*flp1–flp2–tadV–rcpCAB–tadZABCDEFG)* of which 12 are essential for fibril secretion (all except *flp2*, which does not seem to be expressed and *rcpB* which could not be fully assessed due to methodological limitations) and, therefore, the adherence phenotype (Kachlany et al., 2000, 2001; Planet et al., 2003). The *tad* gene locus appears to be present in many bacterial and archaeal genera and can be both chromosome and plasmid borne, indicating a propensity for horizontal gene transfer and is thus often termed the Widespread Colonization Island (Planet et al., 2003; Torres-Monroy and Ullrich, 2018). The TadA protein functions as an ATPase and provides the energy for Flp pilus assembly. Mutations in conserved regions of TadA abolished Flp-pilus production and disrupted the adherence phenotype in *A. actinomycetemcomitans* (Bhattacharjee et al., 2001). We selected the *tadA* gene to examine its taxonomic distribution in the Tara Oceans dataset as a proxy for the distribution of the entire cluster in marine microbes. An HMM profile (Supplementary File 3) constructed from 114 *tadA* genes (including those found in our *Rhodobacterales* MAGs) was searched against the Tara Oceans database. Apart from Archaea, Roseobacters were the largest group of prokaryotes to possess the *tadA* gene and by extension, the *tad* locus, illustrative of the likely importance of these genes in marine Roseobacters and their ability to colonize surfaces (Supplementary Figure 6).

**FIGURE 7:**
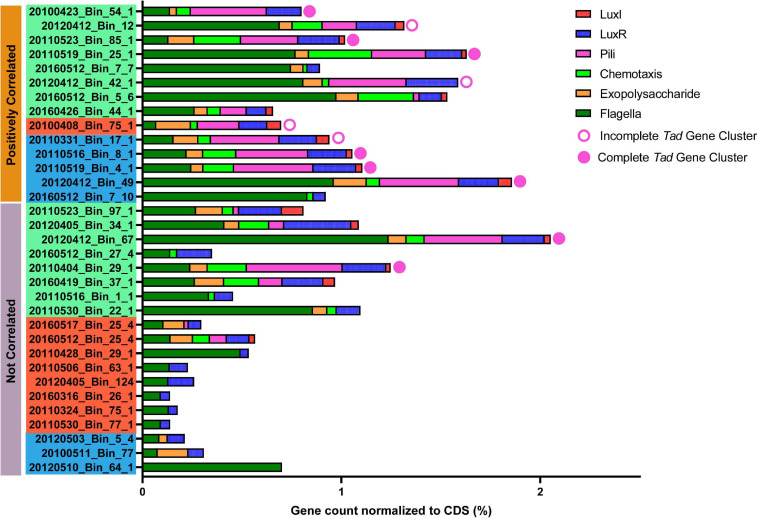
Presence of genes/loci considered important for phycosphere colonization in *Rhodobacterales* MAGs. Gene counts were normalized against the number of coding sequences (CDS) in the respective MAGs. Solid circles indicate a complete *tad* gene locus, open circles indicate near-complete *tad* gene locus (Bins: 20120412_Bin_12, 20120412_Bin_42_1 and 20110331_Bin_17_1 are each missing 1 gene while 2010408_Bin_75_1 is missing 5 genes when compared to MAGs with a complete complement of *tad* genes), while the absence of circles indicate the complete absence of *tad* genes. Positively correlated MAGs are considered putative phycosphere colonizers and non-correlated MAGs are considered putative generalists as established in Figure 5. Color coding of MAG names indicate the MAG grouping based in Figure 3.

The *Rhodobacterales* MAGs were subsequently mined for the presence of a complete *tad* system and other genes that may be important for phycosphere colonization (Figure 7 and Supplementary Figure 7). When compared against the *A. actinomycetemcomitans tad* cluster, more than 70% of the putative phycosphere colonizer MAGs possessed pili genes and complete or nearly complete *tad* gene loci, significantly more (*t*-test, *p* value < 0.01) than putative generalists of which there were only two MAGs from Group I that possessed the complete locus. In general, all the MAGs possessed genes related to motility and 69% of *Rhodobacterales* MAGs possessed genes related to chemotaxis and were significantly present in colonizer MAGs (*t*-test, *p* value < 0.05). These features enable bacteria to sense local concentrations of metabolites and mobilize accordingly in the direction of the source (Miller et al., 2004; Stocker, 2012; Smriga et al., 2016), in this case, phytoplankton derived DOM. Motility and chemotaxis appear to be prerequisites for attachment and biofilm development in a number of Roseobacters (Miller and Belas, 2006; Bruhn et al., 2007). Thus, chemotaxis and motility enable bacteria to enter the phycosphere in response to algal exudates with some individuals switching from a motile lifestyle to a sessile one by attaching to phytoplankton cells (Geng and Belas, 2010). Given the distribution of chemotaxis proteins among the *Rhodobacterales* MAGs, it is likely that a subset of these MAGs may be able to mobilize toward phytoplankton exudates. The molecular mechanism that underpins the switch from a motile to sessile lifestyle has been attributed to the production of, and response to, QS molecules (Fei et al., 2020). QS molecules, typically AHLs produced by Roseobacters, are known to regulate production of antimicrobials, biofilm formation, motility, virulence and nutrient acquisition (Gram et al., 2002; Hmelo et al., 2011; Buchan et al., 2014; Gardiner et al., 2015). Interestingly, there is a higher prevalence of *luxI* homologs in putative phycosphere colonizers (71.4%) than in generalists (31.6%), while *luxR* homologs are more widespread with many MAGs possessing multiple *luxR* genes. This multiplicity in *luxR* homologs allow for bacteria to respond to a wide range of AHLs from other bacteria (Yao et al., 2006; Janssens et al., 2007), while some studies have noted that these so-called solo-*luxR* genes, that are missing their corresponding *luxI* genes, exhibit loss of QS function (Subramoni and Venturi, 2009). Gardiner et al. (2015) reported that a loss of QS function in Roseobacter group member, *Nautella italica* R11 led to an inability to form biofilms or attach to red macroalga. Thus, a subset of MAGs that present with chemotactic, QS and adherence genes may be well suited to colonizing the phycosphere.

Interestingly, Roseobacter isolates lacking *flp* pilus genes were also reported to lack genes encoding proteins for quorum sensing and secondary metabolism (Voget et al., 2015). It is, therefore, likely that putative generalists that lack both *tad* loci and QS genes are unable to colonize the phycosphere and will presumably use constant motility and chemotaxis to acquire DOM from algal cells on an ‘as needed basis’. In contrast, putative phycosphere colonizers should be able to form tight, consistent associations with phytoplankton in the phycosphere using motility and chemotaxis to find phytoplankton cells, then QS, the *tad* gene locus and biofilm formation genes to colonize the phycosphere.

Few flagella related proteins of putative generalist origin were found in the metaproteome, while proteins related to adherence and Flp pili were not detected. This was expected assuming the low relative abundance of putative phycosphere colonizers (Figures 4, 5), the low molecular weight and fragility of pili as well as the general propensity of adhesion related proteins to be in lower abundance than those for motility (Christie-Oleza et al., 2012). There is likely a regulatory link between QS and the *tad* locus, which is presumably a feature that enables specific Roseobacters to attach to algal cells. *Mesorhizobium tianshanense* was shown to possess a LuxR-LuxI type QS system that played a role in adherence to plant root hairs and nodule formation and thus has a critical role in symbiosis (Zheng et al., 2006), while several studies report that in addition to virulence genes, QS circuits modulate *tad* gene expression in *P. aeruginosa* (Schuster et al., 2003; Wagner et al., 2003; Juhas et al., 2005). Additionally, *in vitro* expression studies have also reported that *Marinobacter adhaerens* HP15, which preferentially attaches to the diatom *Thalassiosira weissflogii* expresses several genes related to chemotaxis and attachment, including Flp pili when co-cultured with the diatom (Torres-Monroy and Ullrich, 2018). The model Roseobacter, *Dinoroseobacter shibae* possesses a QS regulatory network in which *tad* pilus genes are modulated by the perception of AHLs (Koppenhofer et al., 2019). Finally, a survey of the genomes of *Sulfitobacter pseudonitzschiae* F5, *Phaeobacter* sp. F10 and *Alteromonas macleodii* F12 which were isolated from the microbiome of diatom *Asterionellopsis glacialis* revealed that *tad* loci are only present in the two Roseobacter genomes (Supplementary Figure 7) but completely absent in the putative opportunist, *A. macleodii* F12, which correlated with the ability of the two Roseobacter species to colonize the phycosphere of this diatom, but not *A. macleodii* F12. Despite the presence of other attachment genes in *A. macleodii* F12, this isolate lacks a complete QS system and thus only the Roseobacters were capable of AHL production, which was shown to modulate their motility and attachment (Fei et al., 2020).

All evidence thus far consistently points toward niche differentiations among algal-associated Roseobacters. The trophic strategy of secreting a higher abundance and diversity of transporter systems can provide an advantage during phytoplankton blooms by enabling utilization of many DOM metabolites (Christie-Oleza et al., 2012). The distribution of Roseobacter transport proteins in the metaproteome supports this as more than 80% of the Roseobacter transport proteins are produced by the putative Roseobacter generalists (Supplementary Figure 8), which were MAGs that had consistent higher relative abundance (Figure 4). Putative phycosphere colonizers do not exhibit this type of growth pattern as their relative abundances rise and fall with particular diatom species. Given their ability to attach this indicates that physical associations could be an alternate trophic strategy to rapid and abundant growth. Shifts in low abundance MAGs relative abundance patterns points toward potential interactions changing over the course of algal bloom cycles. Indeed, this was observed during dinoflagellate blooms where distinct bacterial taxa colonized algal cells during different phases of the bloom (Mayali et al., 2011). Altogether, these findings suggest that while *Rhodobacterales* generally respond to diatom blooms, only a subset of members are able to form close associations and colonize diatom phycospheres.

## Conclusion

The dominance of *Rhodobacterales* in marine environments and their apparent interactions with phytoplankton suggest they possess unique metabolic machinery to access phytoplankton exudates. Metagenomics and metaproteomics provide a unique, culture-independent opportunity to investigate microbial interactions by identifying known genes of importance for interactions with phytoplankton. These tools enable the identification of genes related to biochemical pathways that could be used to compare metabolic and functional gene distribution in microbial communities that occupy different environments or niches (DeLong and Karl, 2005). Investigating these genes and biochemical pathways provide us with a glimpse of how widely distributed these genes and pathways are and how different bacterial lineages may rely on them to interact with phytoplankton. Here we report on comprehensive comparative genomics and metaproteomics of diatom bloom associated members of the order *Rhodobacterales* and provide insight into the functional and metabolic potential of these individuals to colonize diatom phycospheres. Our findings confirm that Roseobacters are among the most important taxa that respond to phytoplankton blooms. More importantly, we show that only a subset of *Rhodobacterales* display strong positive correlations to dominant diatom species during these blooms and that they may use a unique gene cluster, *tad*, in combination with QS, motility and chemotaxis to find and colonize the phycosphere, in strong contrast to putative *Rhodobacterales* generalists that lack the *tad* genes. Future culture-independent work on larger planktonic size fractions, and with Roseobacter isolates, is thus required to investigate the role of these *tad* genes in phycosphere colonization.

## Data Availability Statement

Publicly available datasets were analyzed in this study. This data can be found here: The metagenomic dataset PRJEB28156 for this study can be found in the European Nucleotide Archive under accession [https://www.ebi.ac.uk/ena/data/view/PRJEB28156]. The metaproteomic spectrometric data ID PXD008238 for this study can be found in the PRIDE.

## Author Contributions

AI, RA, and SA conceived the study. BF assembled the metagenomes and binned the MAGs. AI developed and carried out supporting algorithms, bioinformatic analyses and computational pipelines, analyzed the data, and generated figures. AI and SA wrote the manuscript with input from all authors.

## Conflict of Interest

The authors declare that the research was conducted in the absence of any commercial or financial relationships that could be construed as a potential conflict of interest.

## Publisher’s Note

All claims expressed in this article are solely those of the authors and do not necessarily represent those of their affiliated organizations, or those of the publisher, the editors and the reviewers. Any product that may be evaluated in this article, or claim that may be made by its manufacturer, is not guaranteed or endorsed by the publisher.

## Footnotes

1
http://github.com/geronimp/enrich

2
https://www.genome.jp/kegg/ko.html

3
https://merenlab.org/2016/11/08/pangenomics-v2/

4
http://tara-oceans.mio.osupytheas.fr/ocean-gene-atlas/

5
https://github.com/wilkox/gggenes
