# Supplementary material for: Tight Adherence (Tad) Pilus Genes Indicate Putative Niche Differentiation in Phytoplankton Bloom Associated Rhodobacterales
Source: Front Microbiol. 2021 Aug 10;12:718297. doi: 10.3389/fmicb.2021.718297 (PMC8383342; doi:10.3389/fmicb.2021.718297)
Supplement: Supplementary file 3 [file Data_Sheet_3.docx]

# Supplementary Figures

#
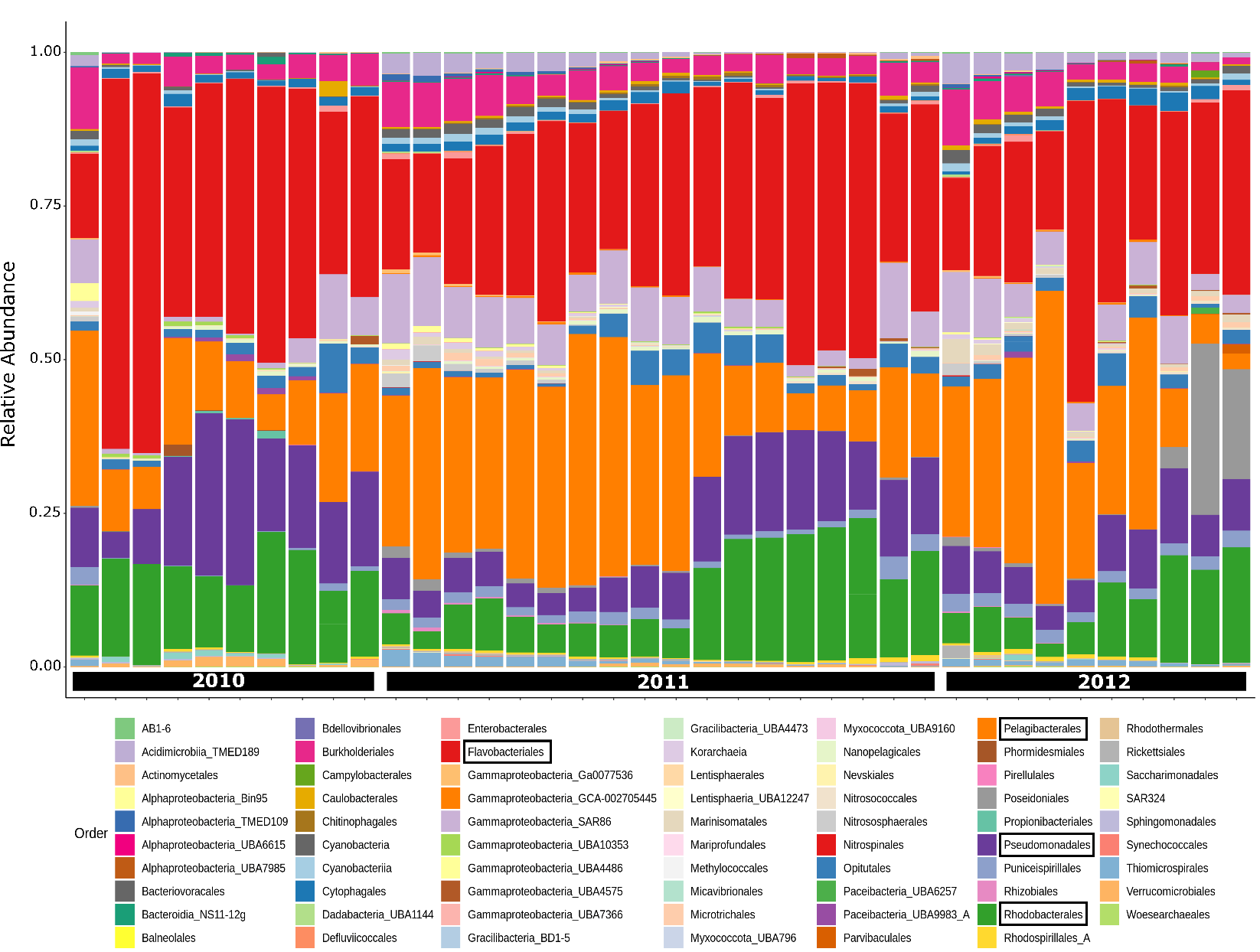


**Supplementary Figure S1. Relative abundance of MAGs during spring phytoplankton blooms in the North Sea.** Metagenome raw reads were mapped to these MAGs at 97% identity. Read counts were normalized against MAG length and the relative abundance calculated. MAG taxonomy is reported at the order level. Each bar within a year represents a single metagenome sample taken during the bloom. See **Supplementary Table S1** for exact dates of metagenomes and dates for which proteome samples were taken. The names of the four most abundance order are boxed.


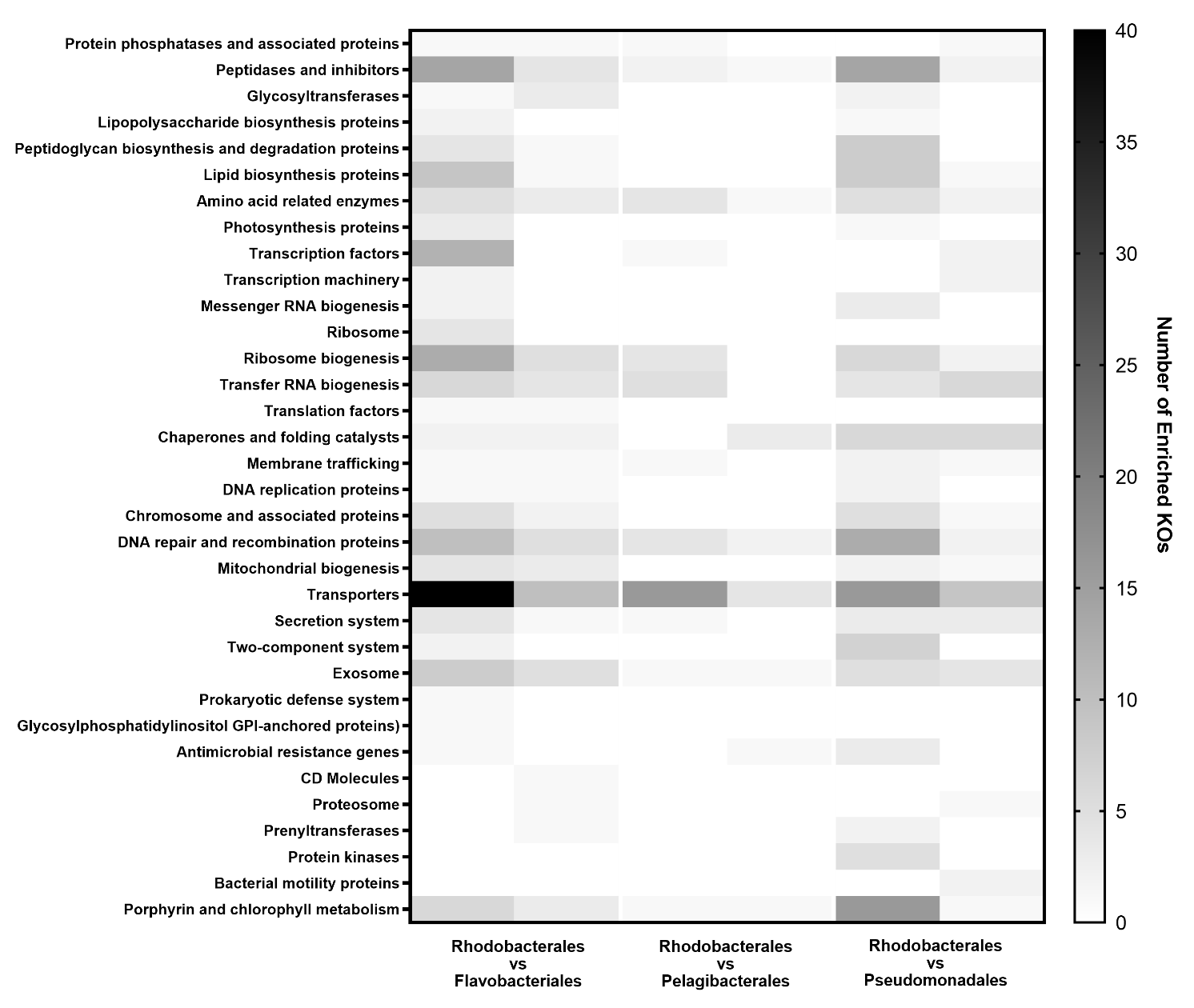


**Supplementary Figure S2. Enriched KEGG Orthology (KO) categories of *Rhodobacterales,*** ***Flavobacteriales*, *Pelagibacterales* and *Pseudomonadales* MAGs**. Representative MAGs (*Rhodobacterales* (33 MAGs), *Flavobacteriales* (135 MAGs), *Pelagibacterales* (8 MAGs) and *Pseudomonadales* (58 MAGs)) were annotated to obtain KEGG Orthology (KO) annotations. KEGG module completion and enrichment of KEGG modules in the top four most abundant orders was determined. Presence/absence of KOs and overrepresentation of KOs within a group was determined and refined based on both significance (corrected *p* value < 0.05) and presence of the KO in >50% of MAGs within an order (**Supplementary File 1**). The final KO annotations per order were searched against the KO database (<https://www.genome.jp/kegg/ko.html>) to obtain the number of enriched KOs that belong to specific BRITE hierarchies. Each pair of columns represent the number of enriched KOs in *Rhodobacterales* MAGs (first column) compared to *Flavobacteriales*, *Pelagibacterales* or *Pseudomonadales* MAGs (second column among the pairs).


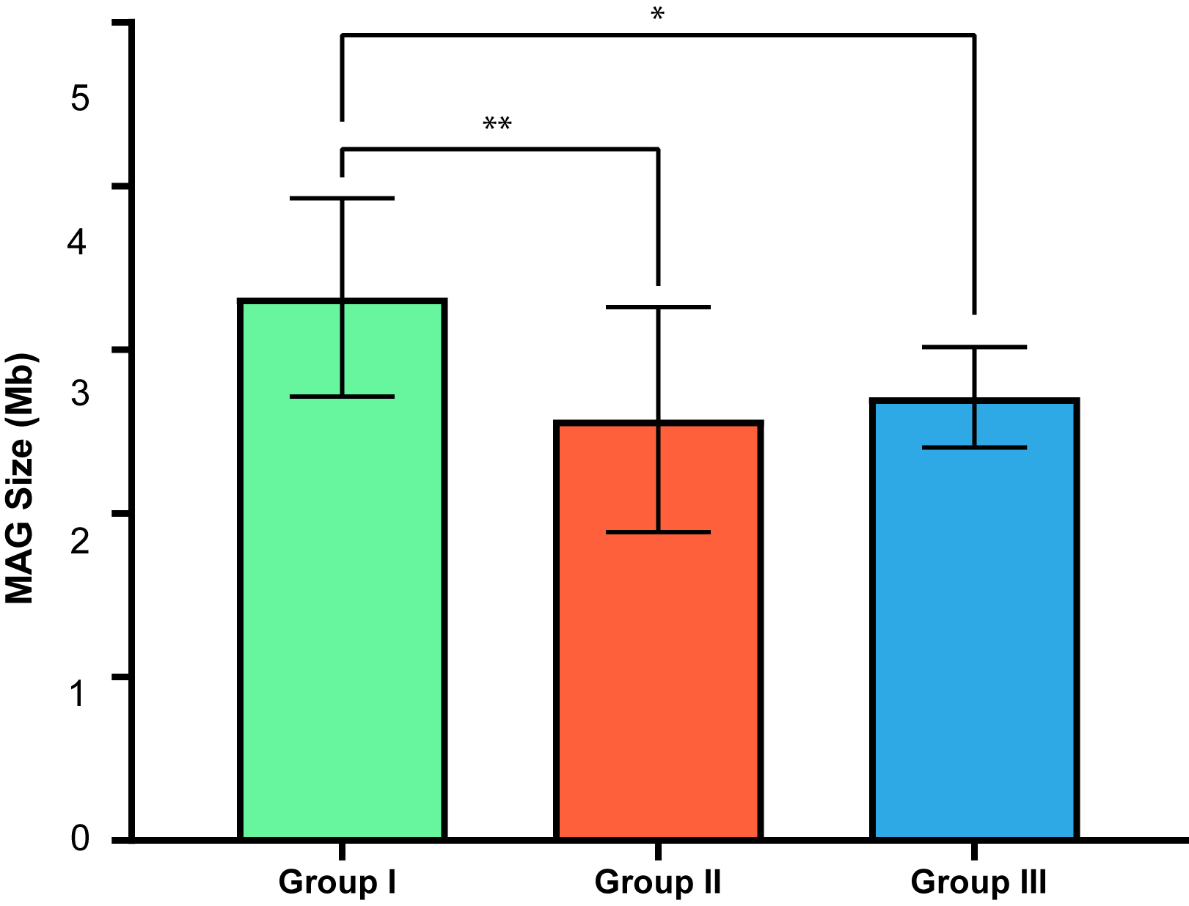


**Supplementary Figure S3. Average *Rhodobacterales* group MAG size.** Groups are designated according to Figure 4. Group I MAGs have a statistically significant larger genome on average as determined by *t*-test (**p* < 0.05 and ***p* < 0.01). The average completeness of MAGs from Group I, II and III are 91.5%, 90.3% and 86.2%, respectively, with <10% contamination which indicates they are of at least medium quality according to Bowers et al. (2017). MAGs details are available in **Supplementary Table S3**.


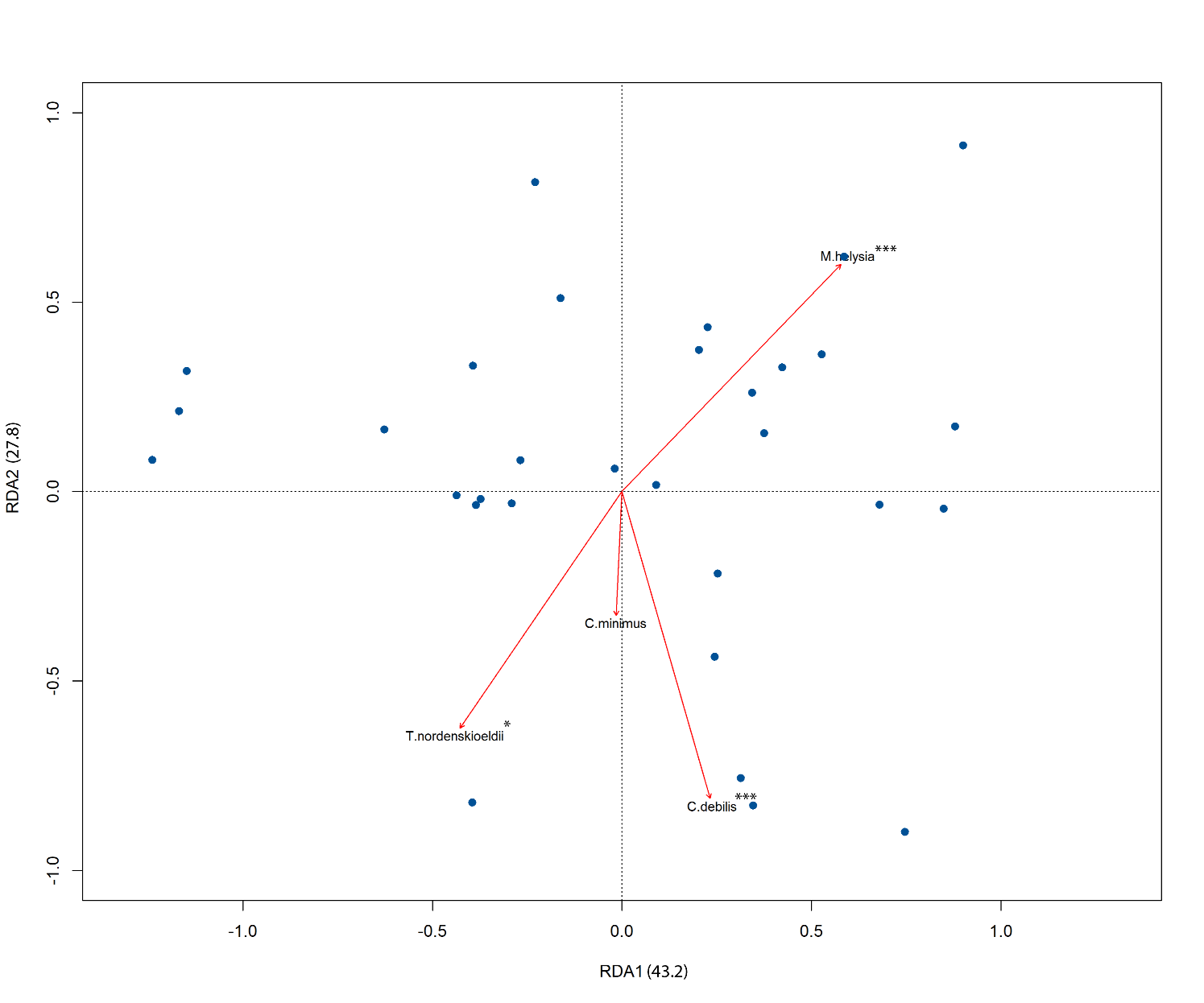
A


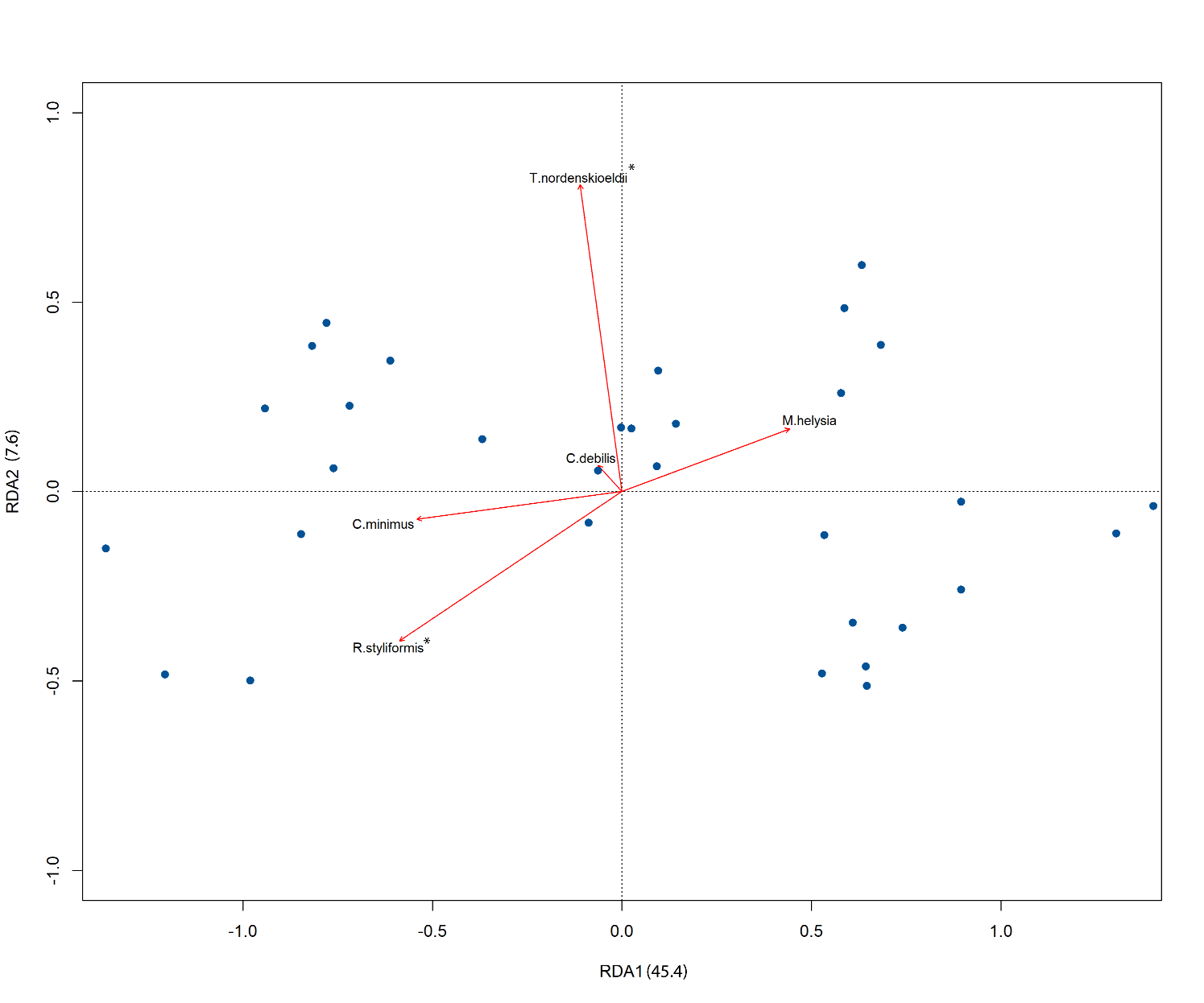
B


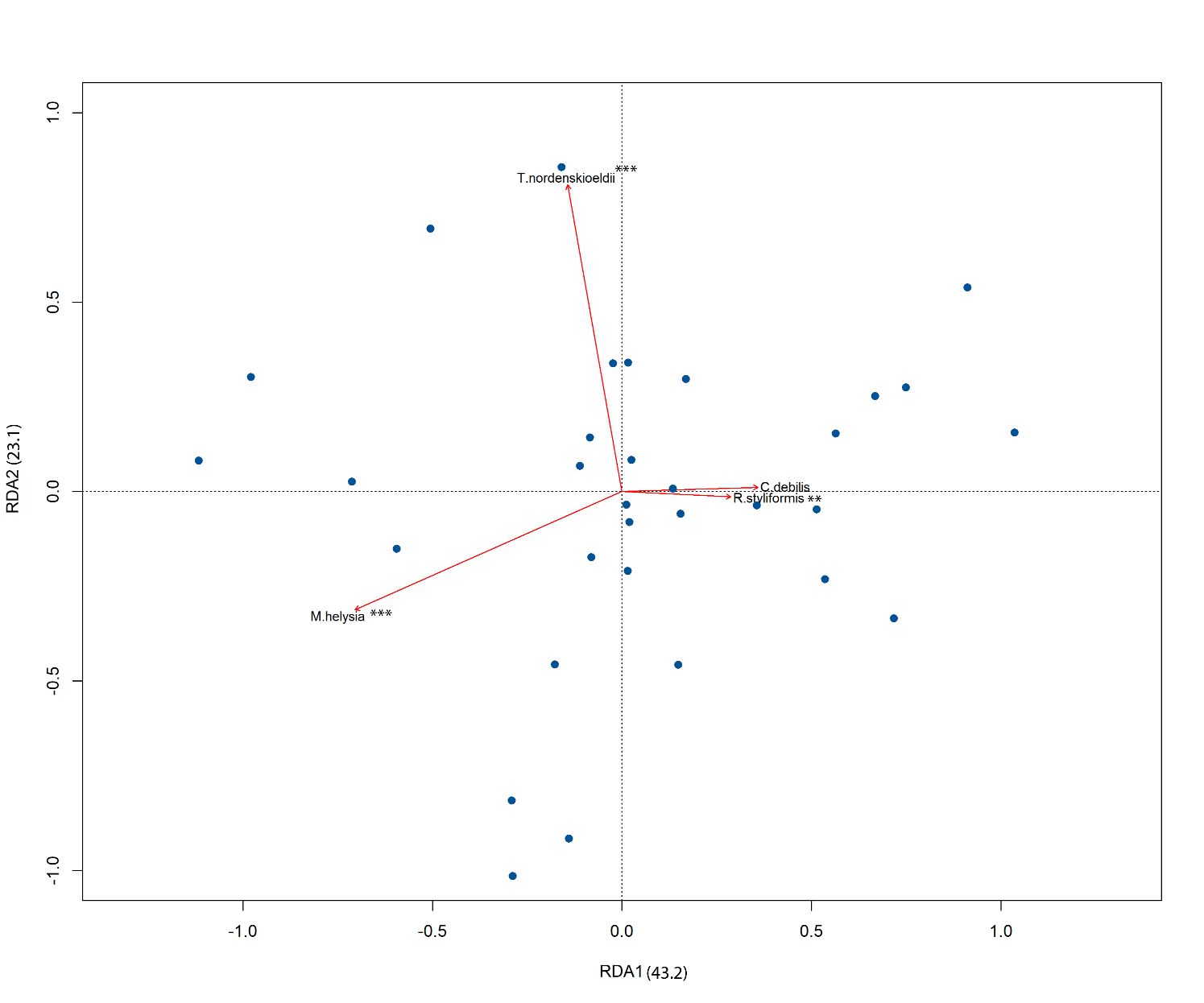


C

**Supplementary Figure S4. Redundancy analysis (RDA) ordination of Roseobacter MAGs elucidates their relationships with diatom species across blooms. (A) 2010; (B) 2011; (C) 2012.** The horizontal (RDA1) and vertical (RDA2) axes capture 71%, 53% and 66% of the variation in MAG relative abundance in 2010, 2011 and 2012, respectively, and were significant in all years: 2010 (Monte Carlo Permutation test, n = 999; F = 6.05; *p* = 0.001), 2011 (Monte Carlo Permutation test, n = 999; F = 2.75; *p* = 0.029) and 2012 (Monte Carlo Permutation test, n = 999; F = 4.99; *p* = 0.001). Red arrows indicate the explanatory variables (diatom species abundance) while blue dots represent the Roseobacter MAGs. The perpendicular distance from a MAG to an arrow is indicative of the relationship, with shorter distances indicating a closer correlation. The statistical significance of the diatom species in explaining the variability in MAG abundance is indicated by asterisks (**p* < 0.05; ***p* = 0.01; ****p* = 0.001).


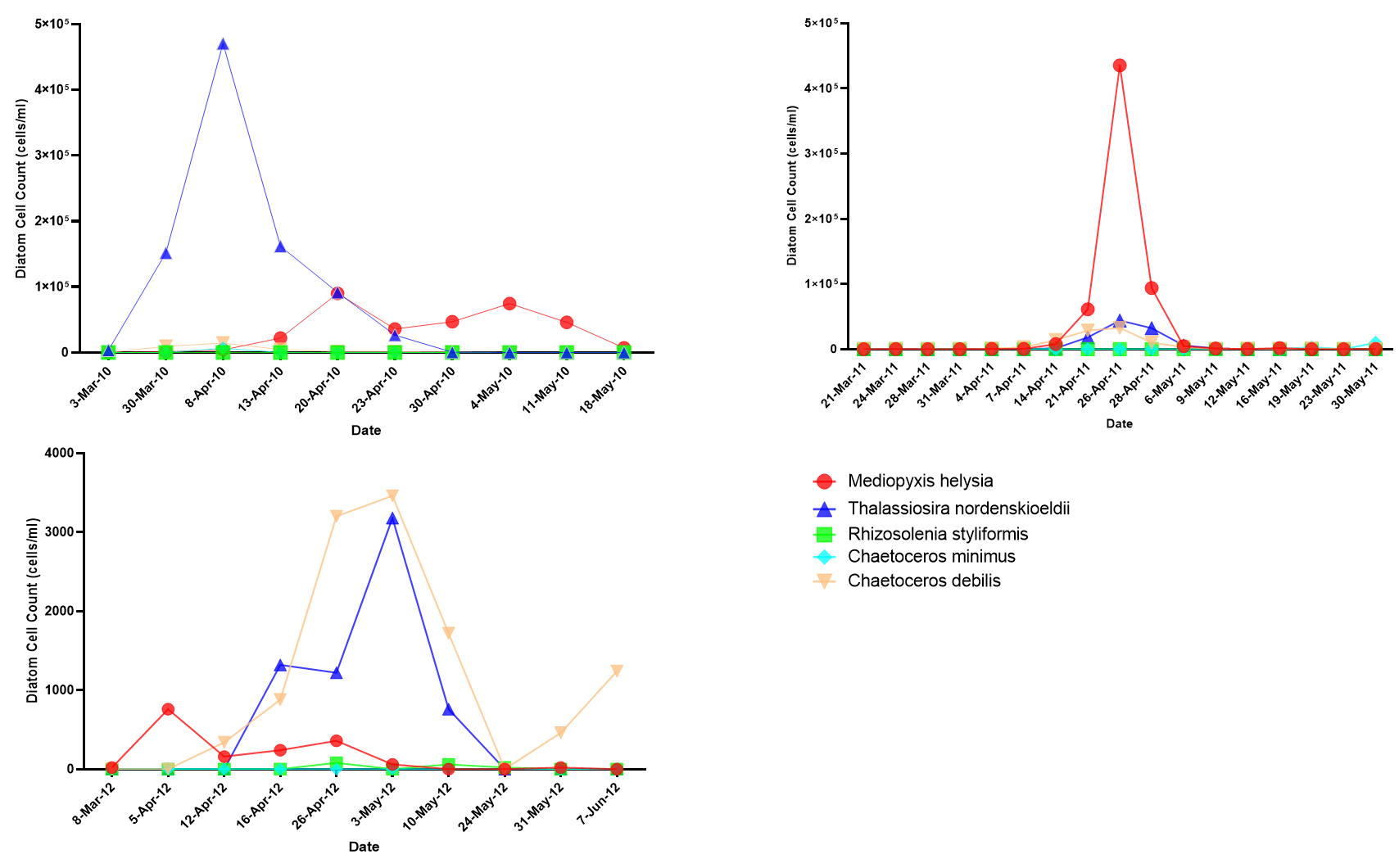


**Supplementary Figure S5. Cell counts of specific diatom species during spring bloom in 2010-2012.** Years 2010 and 2011 were dominated by diatoms while 2012 was dominated by a silicoflagellate species (not shown). Counts were conducted microscopically from surface samples as described in Teeling et al. (2016).


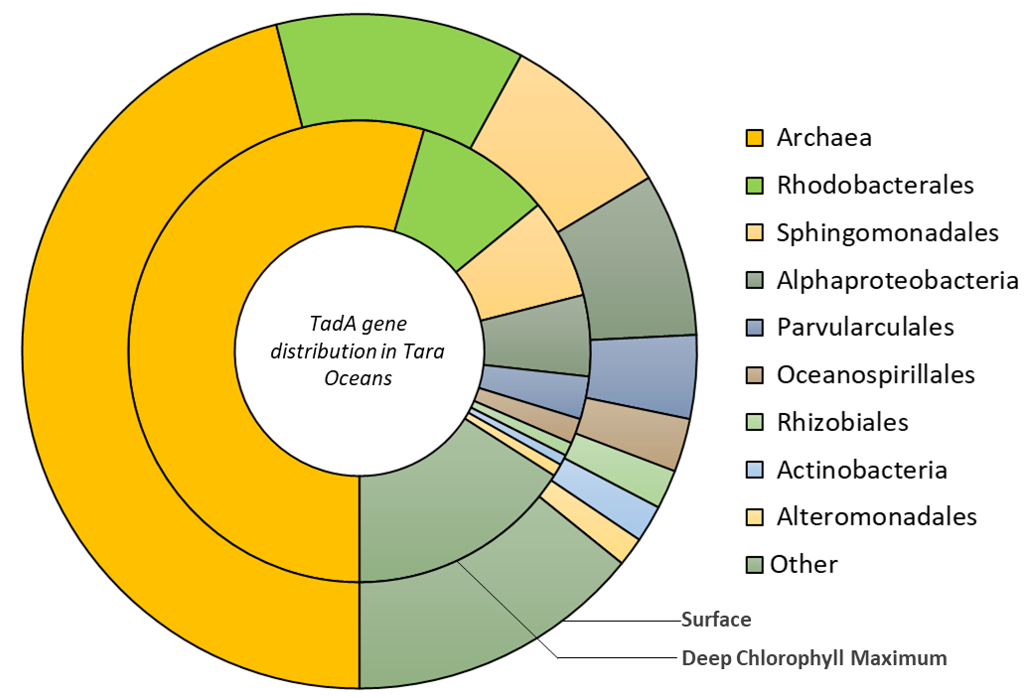


**Supplementary Figure S6. *tadA* gene taxonomic distribution in marine ecosystems.** The *tadA* gene was used as a proxy for the presence of the entire *tad* gene cluster. Abundance of *tadA* gene was determined by querying a *tadA* HMM profile (**Supplementary File 3**) against the Tara Oceans Microbiome Reference Gene Catalog version 1 on the Ocean Gene Atlas (http://tara-oceans.mio.osupytheas.fr/ocean-gene-atlas/) webserver.


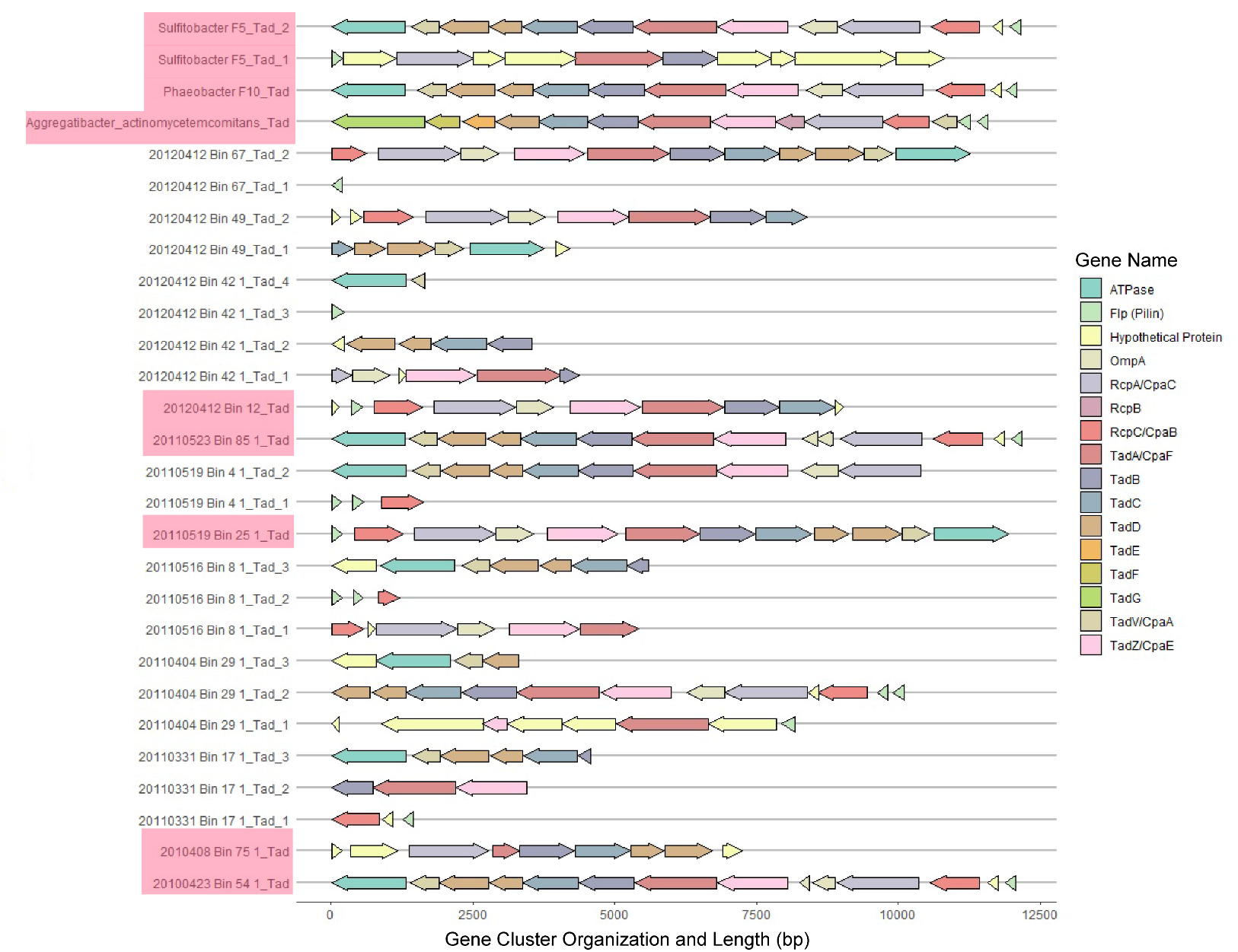


**Supplementary Figure S7. *tad* gene loci presence and organization in *Rhodobacterales* MAGs and genomes.** Completion of the gene cluster was determined by comparison to the *tad* cluster in *Aggregatibacter actinomycetemcomitans* after annotation through RAST. The apparent presence of multiple incomplete *tad* clusters in some MAGs is a result of the locus being located on different contigs; however, these MAGS still possess a full complement of *tad* associated genes as illustrated in Figure 8. Highlighted genome and MAG names indicate the presence of *tad* genes on a single contig.

**Supplementary Figure S8. Distribution of *Rhodobacterales* transport proteins among putative generalist and phycosphere colonizers.** Putative generalists are indicated in greyscale while putative phycosphere colonizers are represented in color. Bars are stacked in descending order as listed in the legend. The majority of transport proteins are produced by the putative generalists.

**Supplementary Materials Reference List**

Bowers, R.M., Kyrpides, N.C., Stepanauskas, R., Harmon-Smith, M., Doud, D., Reddy, T., et al.

(2017). Minimum information about a single amplified genome (MISAG) and a metagenome-assembled genome (MIMAG) of bacteria and archaea. *Nat. Biotechnol.* 35, 725-731. doi: 10.1038/nbt.3893

Teeling, H., Fuchs, B.M., Bennke, C.M., Krüger, K., Chafee, M., Kappelmann, L., et al. (2016). Recurring patterns in bacterioplankton dynamics during coastal spring algae blooms. *Elife* 5**,** e11888. doi: 10.7554/eLife.11888
