# Supplementary material for: Tight Adherence (Tad) Pilus Genes Indicate Putative Niche Differentiation in Phytoplankton Bloom Associated Rhodobacterales
Source: Front Microbiol. 2021 Aug 10;12:718297. doi: 10.3389/fmicb.2021.718297 (PMC8383342; doi:10.3389/fmicb.2021.718297)
Supplement: Supplementary file 1 [file Supplementary_File_2.pdf › Supplementary_File_2.pdf]

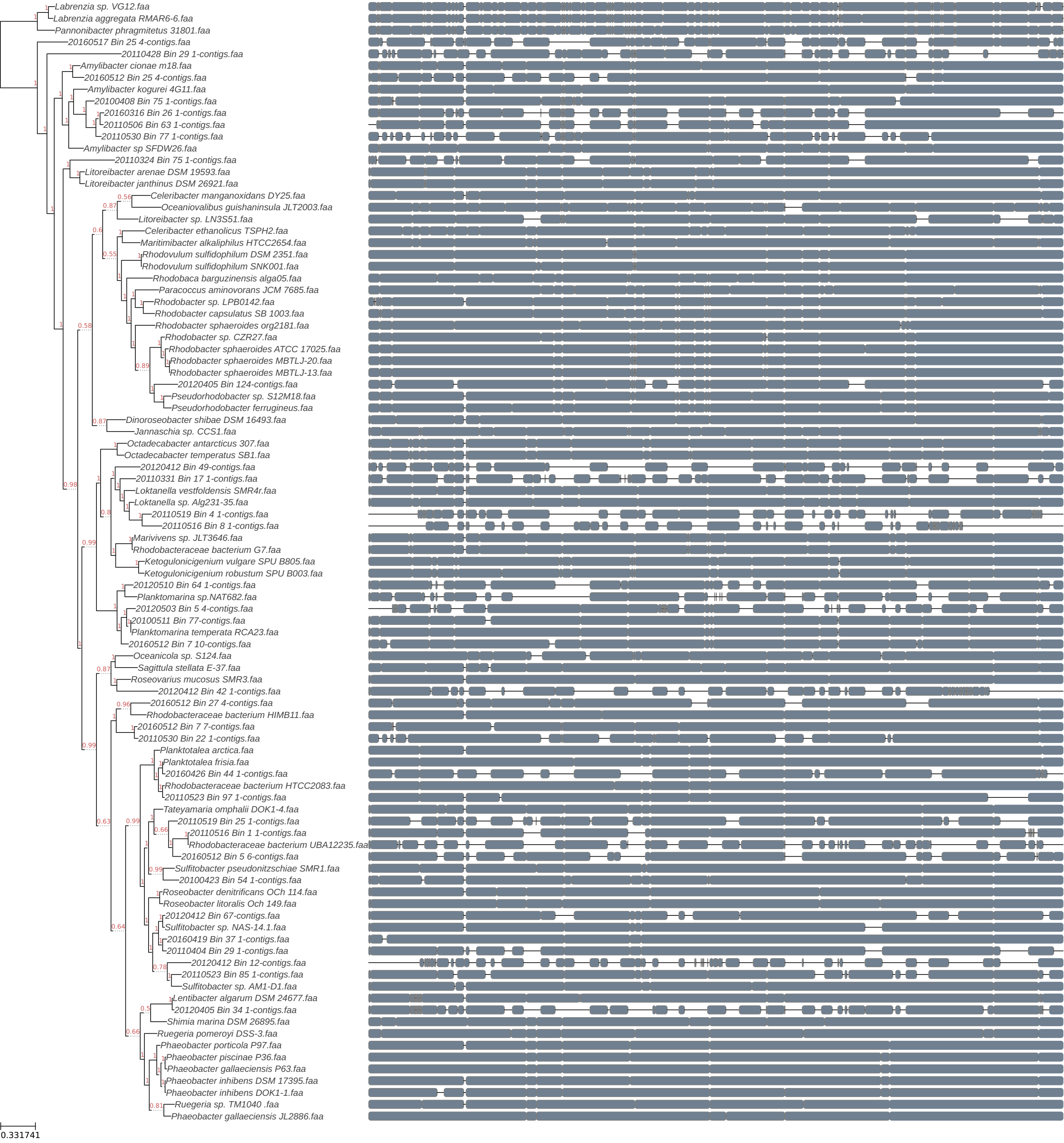

Labrenzia sp. VG12.faa
Labrenzia aggregata RMAR6-6.faa
Pannonibacter phragmitetus 31801.faa
20160517 Bin 25 4-contigs.faa
20110428 Bin 29 1-contigs.faa
Amylibacter cionae m18.faa
20160512 Bin 25 4-contigs.faa
Amylibacter kogurei 4G11.faa
20100408 Bin 75 1-contigs.faa
20160316 Bin 26 1-contigs.faa
20110506 Bin 63 1-contigs.faa
20110530 Bin 77 1-contigs.faa
Amylibacter sp SFDW26.faa
20110324 Bin 75 1-contigs.faa
Litoreibacter arenae DSM 19593.faa
Litoreibacter janthinus DSM 26921.faa
Celeribacter manganoxidans DY25.faa
Oceaniovalibus guishaninsula JLT2003.faa
Litoreibacter sp. LN3S51.faa
Celeribacter ethanolicus TSPH2.faa
Maritimibacter alkaliphilus HTCC2654.faa
Rhodovulum sulfidophilum DSM 2351.faa
Rhodovulum sulfidophilum SNK001.faa
Rhodobaca barguzinensis alga05.faa
Paracoccus aminovorans JCM 7685.faa
Rhodobacter sp. LPB0142.faa
Rhodobacter capsulatus SB 1003.faa
Rhodobacter sphaeroides org2181.faa
Rhodobacter sp. CZR27.faa
Rhodobacter sphaeroides ATCC 17025.faa
Rhodobacter sphaeroides MBTLJ-20.faa
Rhodobacter sphaeroides MBTLJ-13.faa
20120405 Bin 124-contigs.faa
Pseudorhodobacter sp. S12M18.faa
Pseudorhodobacter ferrugineus.faa
Dinoroseobacter shibae DSM 16493.faa
Jannaschia sp. CCS1.faa
Octadecabacter antarcticus 307.faa
Octadecabacter temperatus SB1.faa
20120412 Bin 49-contigs.faa
20110331 Bin 17 1-contigs.faa
Loktanella vestfoldensis SMR4r.faa
Loktanella sp. Alg231-35.faa
20110519 Bin 4 1-contigs.faa
20110516 Bin 8 1-contigs.faa
Marivivens sp. JLT3646.faa
Rhodobacteraceae bacterium G7.faa
Ketogulonicigenium vulgare SPU B805.faa
Ketogulonicigenium robustum SPU B003.faa
20120510 Bin 64 1-contigs.faa
Planktomarina sp.NAT682.faa
20120503 Bin 5 4-contigs.faa
20100511 Bin 77-contigs.faa
Planktomarina temperata RCA23.faa
20160512 Bin 7 10-contigs.faa
Oceanicola sp. S124.faa
Sagittula stellata E-37.faa
Roseovarius mucosus SMR3.faa
20120412 Bin 42 1-contigs.faa
20160512 Bin 27 4-contigs.faa
Rhodobacteraceae bacterium HIMB11.faa
20160512 Bin 7 7-contigs.faa
20110530 Bin 22 1-contigs.faa
Planktotalea arctica.faa
Planktotalea frisia.faa
20160426 Bin 44 1-contigs.faa
Rhodobacteraceae bacterium HTCC2083.faa
20110523 Bin 97 1-contigs.faa
Tateyamaria omphalii DOK1-4.faa
20110519 Bin 25 1-contigs.faa
20110516 Bin 1 1-contigs.faa
Rhodobacteraceae bacterium UBA12235.faa
20160512 Bin 5 6-contigs.faa
Sulfitobacter pseudonitzschiae SMR1.faa
20100423 Bin 54 1-contigs.faa
Roseobacter denitrificans OCh 114.faa
Roseobacter litoralis Och 149.faa
20120412 Bin 67-contigs.faa
Sulfitobacter sp. NAS-14.1.faa
20160419 Bin 37 1-contigs.faa
20110404 Bin 29 1-contigs.faa
20120412 Bin 12-contigs.faa
20110523 Bin 85 1-contigs.faa
Sulfitobacter sp. AM1-D1.faa
Lentibacter algarum DSM 24677.faa
20120405 Bin 34 1-contigs.faa
Shimia marina DSM 26895.faa
Ruegeria pomeroyi DSS-3.faa
Phaeobacter porticola P97.faa
Phaeobacter piscinae P36.faa
Phaeobacter gallaeciensis P63.faa
Phaeobacter inhibens DSM 17395.faa
Phaeobacter inhibens DOK1-1.faa
Ruegeria sp. TM1040 .faa
Phaeobacter gallaeciensis JL2886.faa
0.331741
